# Activated chemical bonds in nanoporous and amorphous iridium oxides favor low overpotential for oxygen evolution reaction

**DOI:** 10.1038/s41467-022-30838-y

**Published:** 2022-06-08

**Authors:** Sangseob Lee, Yun-Jae Lee, Giyeok Lee, Aloysius Soon

**Affiliations:** Department of Materials Science & Engineering and Center for Artificial Synesthesia Materials Discovery, Yonsei University, Seoul, 03722 Republic of Korea

**Keywords:** Materials for energy and catalysis, Theory and computation

## Abstract

To date, the search for active, selective, and stable electrocatalysts for the oxygen evolution reaction (OER) has not ceased and a detailed atomic-level design of the OER catalyst remains an outstanding (if not, compelling) problem. Considerable studies on different surfaces and polymorphs of iridium oxides (with varying stoichiometries and dopants) have emerged over the years, showing much higher OER activity than the conventionally reported rutile-type IrO_2_. Here, we have considered different metastable nanoporous and amorphous iridium oxides of different chemical stoichiometries. Using first-principles electronic structure calculations, we investigate the (electro)chemical stability, intercalation properties, and electronic structure of these iridium oxides. Using an empirical regression model between the Ir-O bond characteristics and the measured OER overpotentials, we demonstrate how activated Ir-O bonds (and the presence of more electrophilic oxygens) in these less understood polymorphs of iridium oxides can explain their superior OER performance observed in experiments.

## Abstract (web summary)

Here the authors use density-functional theory calculations to examine structure-property relations of nanoporous and amorphous iridium oxides and reconcile the superior oxygen evolution reaction catalytic performance reported in previous experiments.

## Introduction

To achieve sustainable energy production, solar-driven (electro)conversion of CO_2_ and H_2_O to value-added solar fuels and O_2_ is a promising means to correct the global carbon balance and provide a sustainable alternative to conventional fossil fuels^1–3^. Here, the anodic reaction—commonly known as the oxygen evolution reaction (OER), is an important half-cell reaction where H_2_O is catalytically split to evolve O_2_. However, due to the intrinsic sluggish kinetics of the OER, this leads to an overall poor catalytic performance in general.

Thus, to improve the long-term efficacy of this anodic reaction, the search for active, selective, and stable OER electrocatalysts has been on the rise, and amongst them, oxides (and oxyhydroxides) of iridium and ruthenium are known for their outstanding stability and reactivity in acidic environments^2,4^. A promising way to tune and engineer the structure–property relations of these oxide catalysts is to control their stoichiometry and polymorphic phase at the atomic-level^5–9^.

Willinger et al. experimentally found that channel-like microstructures (resembling the hollandite- or romanechite-type motifs) contributed to a better OER efficiency and catalytic stability of the amorphous iridium oxide catalyst^10^. It was also reported that the intercalation of alkaline earth metal cations in the nanopores of these channel-type microstructures adds stability to the overall catalyst structure^11–13^. Though these accounts demonstrate improved OER performance over conventional crystalline rutile-structured IrO_2_, a fundamental atomic-scale understanding of these nanopore-containing amorphous iridium oxides is very much lacking. Thus, it greatly hinders the establishment of a design rule for further performance improvement.

In an effort to fill this lack, a very recent high-throughput computational study^14^ (assisted by an generalizable active-learning accelerated algorithm) has attempted to investigate the role of polymorphism in both IrO_2_ and IrO_3_ (and their surfaces) for OER activity. Using a machine-learning-based surrogate model, they were able to rationalize that under OER technical catalysis conditions, the *α*-phase of IrO_3_ was, in fact, predicted to have higher thermodynamic stability and high OER activity, as compared to that of rutile-type IrO_2_. However, nanoporous (i.e. crystal structures containing nanopores and nanochannels) and amorphous iridium oxides—which have been proposed in various experiments^11–13^—have not been included nor examined in this study.

Moreover, from the OER reaction mechanistic point-of-view, there is still an ongoing debate as to whether the direct formation of O_2_ molecule proceeds via the adsorbate evolving mechanism (AEM, where concerted electron–proton transfer steps are involved)^15,16^, or by means of the lattice oxygen mechanism (LOM, where lattice oxygen participates via a Mars van Krevelen-type process)^17^. O isotope-labeling experiments have revealed and suggested that the LOM is more likely on the surface of IrO_2_ where the Mars van Krevelen-type process has also been proposed for non-oxide compounds (e.g. in sulfides and chlorides)^17^. Specifically, for the LOM, it has been proposed in many reports that the presence of electrophilic oxygen on the surface of iridium oxide will play an important role in the adsorption of the nucleophilic H_2_O molecule via the so-called flexible charge state of Ir cations in the different phases of iridium oxide^10,18–21^.

In addition, the chemical characteristics of the Ir–O bond has been suggested as a key descriptor for both O_2_ desorption and the adsorption of H_2_O on iridium oxides^15^. In the same vein, the use of the metal–sulfur bond strength as a descriptor for Mars van Krevelen-type dehydrosulfurization has also been deliberated in a previous report^17^. These suggested atomistic features (e.g. the correlation between the flexibility of the charge state of Ir cations and the presence of electrophilic oxygen, and Ir–O bonding characteristic) are still poorly understood, especially for experimentally observed nanoporous and amorphous iridium oxides.

In this work, to bolster the work of Flores et al. ^14^, we expand and include experimentally motivated IrO_2_ polymorphs (such as nanoporous hollandite-, romanechite-, and todorokite-type IrO_2_ with K-intercalation) as well as amorphous structures of iridium oxides of varying chemical stoichiometry. Here, using first-principles density-functional theory (DFT) calculations, we show that the nonequivalent connectivity in the amorphous iridium oxide structures greatly improves the flexibility of the Ir charge states, and hence promoting the presence of electrophilic oxygens in them, when compared to their crystalline counterparts. We also demonstrate that a Pauling-like relation between the Ir–O bond length versus bond strength for the Ir–O bonds exist in amorphous iridium oxides, corroborating with the proposal of flexible charges states and activated bonds for more efficient OER catalysis. Via an empirical regression model between the Ir–O bond characteristics and the measured OER overpotentials, we propose that these less understood metastable nanoporous and amorphous iridium oxides may indeed afford a lower OER overpotential, reconciling their superior OER catalytic performance in recent experiments.

## Results

### Crystalline and nanoporous phases of iridium oxides

**Fig. 1:**
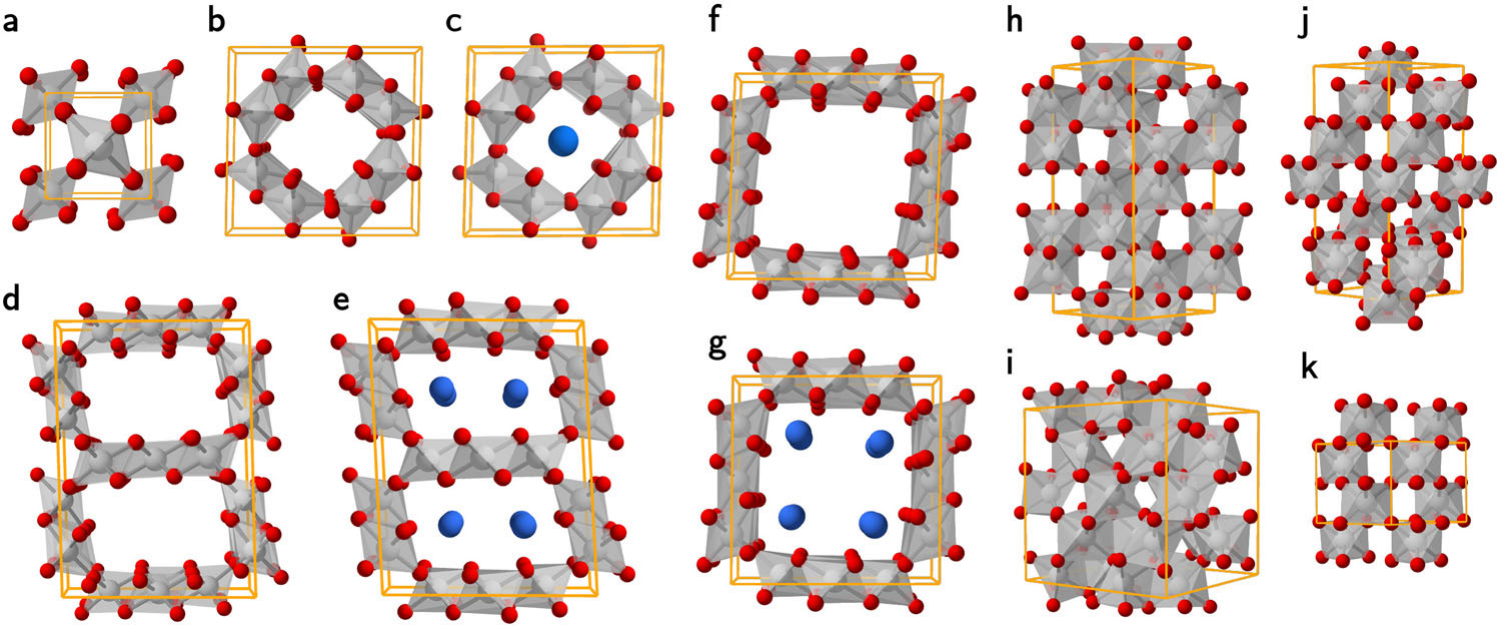
Atomic and crystal structures of the crystalline iridium oxides. **a** Rutile-type R-IrO_2_. **b** Hollandite-type Ho-IrO_2_. **c** K-intercalated hollandite-type 1K + Ho-IrO_2_. **d** Romanechite-type Ro-IrO_2_. **e** K-intercalated romanechite-type 2K + Ro-IrO_2_. **f** Todorokite-type To-IrO_2_. **g** K-intercalated todorokite-type 4K + To-IrO_2_. **h** Corundum-based C-IrO_1.5_. **i** bixbyite-based B-IrO_1.5_. **j** $R\overline{3}c$ R-IrO_3_. **k** *P*6_3_22 P-IrO_3_. The iridium, oxygen, and potassium atoms are depicted as gray, red, and blue spheres, respectively, while the octahedra of IrO_6_ is shaded in gray. The bulk unit cell is represented by the lines in orange.

Following a recent survey of iridium oxide polymorphs by machine learning approaches^14^, we have adopted the low-energy polymorphs of IrO_2_ and IrO_3_—namely, the rutile phase of IrO_2_ (R-IrO_2_; Fig. 1a), and the $R\overline{3}c$ (R-IrO_3_; Fig. 1j) and *P*6_3_22 (P-IrO_3_; Fig. 1k) phases of IrO_3_. In addition, we have also included the experimentally proposed MnO_2_-like nanoporous structures of IrO_2_^10,12,13^ (which were not included in the previous computational studies)—in particular, the hollandite (Ho-IrO_2_), romanechite (Ro-IrO_2_), and todorokite (To-IrO_2_) phases (as presented in Fig. 1b, d, f, respectively). These nanoporous or nanochanneled oxide structures are typically intercalated with alkali metal ions to improve structural stability and have also been proposed to improve OER catalytic activities^10,12^.

To take the ion intercalation into account, in Fig. 1c, e, g, we show the K ion intercalated IrO_2_ hollandite structure (1K + Ho-IrO_2_), the K ion intercalated IrO_2_ romanechite structure (2K + Ro-IrO_2_), and the K ion intercalated IrO_2_ todorokite structure (4K + To-IrO_2_), respectively. To further extend our theoretical investigation on non-stoichiometric iridium oxides, fictitious crystal structures of IrO_1.5_ (or Ir_2_O_3_) have been included in this work—namely the corundum phase (C-IrO_1.5_; Fig. 1h) and the bixbyite phase (B-IrO_1.5_; Fig. 1i).

Using the optB86b *xc* functional, we have computed the optimized lattice parameters (with the corresponding space groups) for all crystalline phases of IrO_2_, IrO_1.5_, and IrO_3_ polymorphs and listed their values in Table 1. In our DFT calculations, the calculated lattice parameters are well within 1–2% agreement with available experimental values (e.g. the experimentally reported *a*, *b*, and *c* lattice constants for rutile R-IrO_2_ are 4.51, 4.51, and 3.16 Å, respectively)^22^. It is worth noting that there has been a previous attempt to experimentally expound on the crystallography of the hollandite-type iridates^23^. For the hollandite-type iridates, our DFT calculations consistently favor the lower symmetry monoclinic *C*2/*m* phase (as opposed to the tetragonal phase), regardless of ion intercalation. We also find that the volumetric changes to the intercalated nanoporous IrO_2_ defer according to channel size. For instance, the volume of 2K + Ro-IrO_2_ shrinks by about 3% while that of 4K + To-IrO_2_ expends by about 5%.

**Table 1:** Calculated lattice parameters, space group, and thermodynamic properties.

Ir–O system	*a* (Å)	*b* (Å)	*c* (Å)	*β* (∘)	Space group	Δ*H*^f^	${{\Delta }}{G}_{300\,{{{{{{{\rm{K}}}}}}}}}^{{{{{{{{\rm{f}}}}}}}}}$	${{\Delta }}{G}_{600\,{{{{{{{\rm{K}}}}}}}}}^{{{{{{{{\rm{f}}}}}}}}}$	Δ*H*^int^
R-IrO_2_	4.51	4.51	3.19	90.0	*P*4_2_/*mnm*	−0.97	−0.94	−1.03	
Ho-IrO_2_	10.24	3.19	9.95	91.0	*C*2/*m*	−0.80	−0.78	−0.88	
1K + Ho-IrO_2_	10.10	3.19	9.77	91.7	*C*2/*m*	−1.01	−0.99	−1.09	−3.52
Ro-IrO_2_	14.11	3.18	9.91	91.4	*C*2/*m*	−0.77	−0.75	−0.85	
2K + Ro-IrO_2_	13.11	3.20	10.24	93.3	*C*2/*m*	−1.03	−1.02	−1.13	−3.01
To-IrO_2_	9.88	3.17	9.79	92.1	*P*2/*m*	−0.74	−0.78	−0.83	
4K + To-IrO_2_	9.99	3.23	9.97	90.5	*P*2/*m*	−1.02	−1.01	−1.11	−2.28
*a*-IrO_2_	10.71	9.83	10.44		*P*1	−0.58	−0.56	−0.65	
C-IrO_1.5_	5.27	13.90	5.27	120.0	$R\overline{3}c$	−0.48	−0.46	−0.56	
B-IrO_1.5_	9.73	9.73	9.73	90.0	$Ia\overline{3}$	−0.37	−0.35	−0.45	
*a*-IrO_1.5_	10.07	9.03	10.37		*P*1	−0.43	−0.41	−0.51	
R-IrO_3_	4.82	12.77	4.82	120.0	$R\overline{3}c$	−0.72	−0.69	−0.77	
P-IrO_3_	4.75	4.35	4.75	120.0	*P*6_3_22	−0.72	−0.69	−0.77	
*a*-IrO_3_	10.94	8.64	11.38		*P*1	−0.48	−0.45	−0.54	

Lattice parameters (*a*, *b*, and *c* are given in Å, and *β* in ^∘^), crystal space group, the formation energy (Δ*H*^f^, given in eV/atom), the Gibbs energy of formation (Δ*G*^f^, given in eV/atom for temperatures 300 and 600 K), and the intercalation energy (Δ*H*_int_, given in eV/K atom) for the various iridium oxide systems.

### Amorphous phases of iridium oxides

Besides the commonly reported crystalline phases of active IrO_*x*_ for OER, recent experimental reports of an amorphous IrO_*x*_ phase could well be responsible for the high OER activity observed^12,20,24^. To generate approximate atomistic models of amorphous IrO_*x*_, we perform *ai*MD calculations (following the melt-and-quench method^25^) for various chemical stoichiometries (henceforth labeled as *a*-IrO_2_, *a*-IrO_1.5_, and *a*-IrO_3_, accordingly) while ensuring numerical convergence with supercell size.

**Fig. 2:**
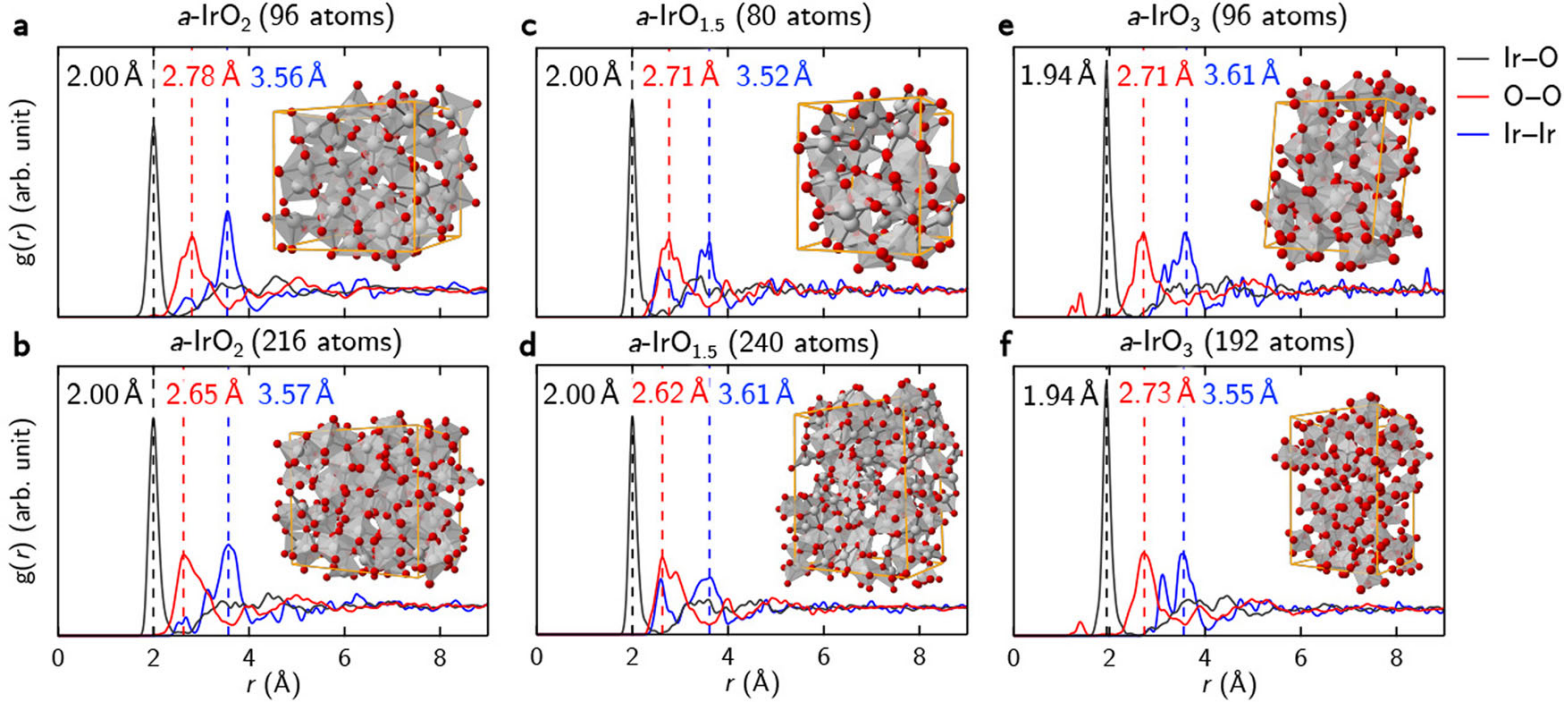
Partial radial distribution function (RDF, *g*(*r*)) for the amorphous phases. **a**, **b** IrO_2_, **c**, **d** IrO_1.5_. and **e**, **f** IrO_3_. The total number of atoms per simulation cell is shown for each case. The highest peak for the Ir–O, O–O, and Ir–Ir bond pair in the *g*(*r*) is denoted by a vertical black, red, and blue dotted line, respectively. The optimized atomic structure for each amorphous iridium oxide phase is also shown, where the gray and red spheres represent the iridium and oxygen atoms, respectively. The simulation cell is represented by the solid lines in orange.

We calculate and plot the calculated partial radial distribution function, *g*(*r*) of Ir–O (in black), O–O (in red), and Ir–Ir (in blue) bond pairs (cf. Supplementary Eq. (1) for *a*-IrO_2_, *a*-IrO_1.5_, and *a*-IrO_3_ in Fig. 2. For all models presented here for amorphous iridium oxide, we do not observe any long-range ordering beyond 4 Å. For *a*-IrO_2_, the representative *g*(*r*) peaks (indicated by the vertical dashed lines; 2.00, 2.78, and 3.56 Å for the Ir–O, O–O, and Ir–Ir bond pairs) are shown in Fig. 2a, agreeing very well with the reported experimental values of 2.00 Å for the Ir–O bond distance^10,12^. When considering the much larger supercell of 216 atoms (in Fig. 2b), we find that the representative *g*(*r*) peaks are somewhat unchanged, and thus lending support that our smaller 96-atom supercell may be appropriate for modeling *a*-IrO_2_. Likewise, for both *a*-IrO_1.5_ (in Fig. 2c, d) and *a*-IrO_3_ (in Fig. 2e, f), similar agreement is met and we can draw the conclusion that amorphous iridium oxide yields an averaged Ir–O, O–O, and Ir–Ir bond distances of 2.0, 2.7, and 3.6 Å, respectively—irregardless of its chemical stoichiometry^26,27^. Incidentally, we note that the very small *g*(*r*) peak for *a*-IrO_3_ near 1.5 Å is attributed to small oxygen clusters in the model.

In addition, the calculated mass densities for *a*-IrO_2_, *a*-IrO_1.5_, and *a*-IrO_3_ are found to be 10.7, 12.2, and 8.9 g/cm^3^, respectively. When compared to the experimentally reported mass density of crystalline IrO_2_ (i.e. 11.7 g/cm^3^),^28^ our calculated value for *a*-IrO_2_ appears somewhat smaller and this is inline with that found for other materials^29^. This difference may be attributed to the lower average coordination number of cations in the amorphous phase when compared to the crystalline phase^30^. Moreover, a weak correlation between our MD-determined mass densities and the oxygen content is also suggested from our calculations^31^.

### Thermodynamic and electrochemical stability

To address the thermodynamic stability for the various iridium oxide structures we considered in this work, we calculate the formation enthalpy, Δ*H*^f^, via Suppplementary Eq. (2) and are tabulated in Table 1. From our calculations, agreeing with previous studies^10,15^, rutile IrO_2_ is found to be the thermodynamic ground state structure for iridium dioxide, while both R-IrO_3_ ($R\overline{3}c$) and P-IrO_3_ (*P*6_3_22) are the representative ground state structures for iridium trioxide (differing only by 1 meV/f.u.). For the hypothetical IrO_1.5_, the corundum phase, C-IrO_1.5_ is taken as the lowest energy structure.

The nanoporous Ho-, Ro-, and To-IrO_2_ are all found to be metastable with respect to rutile IrO_2_ and additional stability is gained upon K intercalation. To analyze the intercalation energetics in these nanoporous iridium oxide structures, we have also calculated their intercalation energy, Δ*H*^int^ (cf. Supplementary Eq. (4)) and are tabulated in Table 1. They are found to be largely exothermic and concur with earlier experimental and theoretical reports where cation intercalation in nanoporous oxides are known to add stability to the overall structure^10,11,13,32,33^. Considering the amorphous analogs to these crystalline iridium oxides, *a*-IrO_2_, *a*-IrO_1.5_, and *a*-IrO_3_ are also determined to be metastable with respect to their crystalline ground state counterparts. Thermodynamic metastability in other amorphous oxides (e.g. Sb_*x*_O_*y*_^26^) are well discussed in literature.

To account for thermal vibration contributions to the overall thermodynamic stability in these iridium oxides, using the Debye model, we estimate their vibrational energy, *F*^vib^ (cf. Supplementary Eq. (3)) and plot the variation of *F*^vib^ with temperature, *T* in the Supplementary Fig. 1. For comparison, we have also included the Gibbs energy of formation, Δ*G*^f^ for *T* = 300 and 600 K in Table 1. We conclude that thermal effects are minimal for the overall thermodynamic stability in these iridium oxides.

**Fig. 3:**
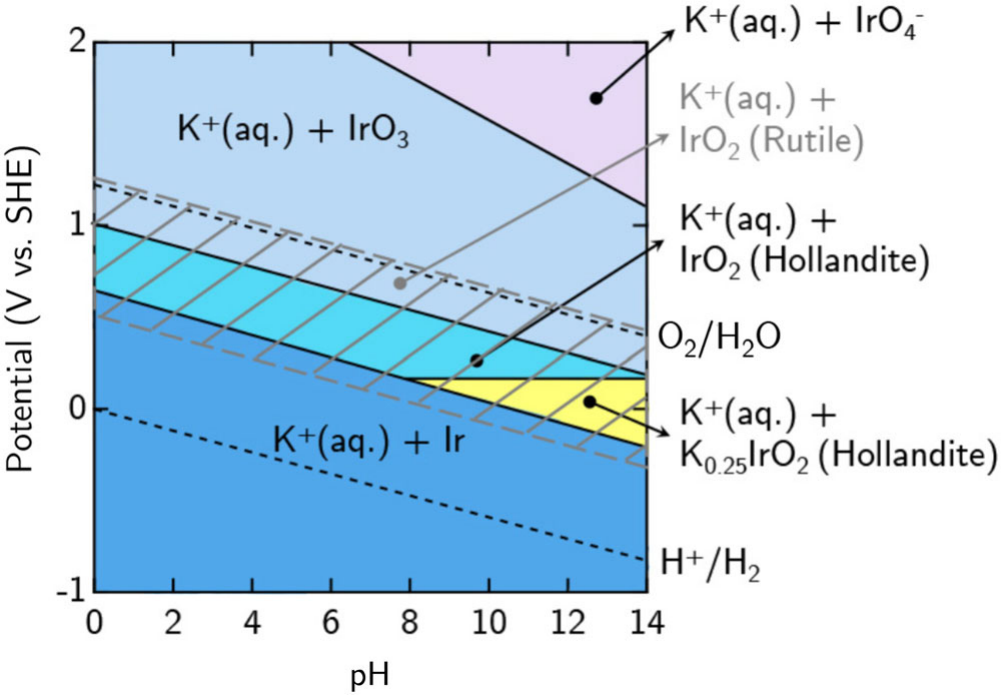
DFT-derived Pourbaix phase diagram for the various iridium oxides and their K-intercalated phases. The shaded region (with gray lines) denotes the stability region for rutile IrO_2_. The standard potentials for water oxidation (O^2−^/H_2_O) and reduction (H^+^/H_2_) are shown as black dotted lines. Following ref. ^10^, the ratio of K and Ir, and the concentration of the ion species are taken as 5:1 and 10^−3^ mol/L, respectively.

Now, to further discuss the stability of these oxides under technical catalysis or synthesis growth conditions, we examine their thermodynamic stability under an electrochemical environment^5,34^. To do this, we construct the DFT-derived Pourbaix phase diagram by considering the reaction energy, Δ*μ* and various relevant ionic species (cf. Supplementary Eq. (5) and Supplementary Table 1) and is presented in Fig. 3. Taking a recent experimental report^10^ as a reference, the ratio of K and Ir, and the concentration of the ion species are taken as 5:1 and 10^−3^ mol/L, respectively.

Within the considered range of pH and the electrode potentials in Fig. 3, marked as the shaded region (with gray lines), the thermodynamic ground state R-IrO_2_ is predicted to be stable under low applied potentials and under both acidic and basic conditions. This is in accord with previous reports where the rutile IrO_2_ phase exhibits good electrochemical stability^14^. However, under certain growth conditions that kinetically hinder the formation of the rutile phase, it is interesting to find that the metastable nanoporous Ho-IrO_2_ phase can be synthesized within a relatively narrow window of stability (as in the turquoise shaded region in Fig. 3). More importantly, under more basic conditions as shown in the yellow shaded region of Fig. 3, 1K + Ho-IrO_2_ (K_0.25_IrO_2_) may form when assuming the kinetic hinderance of R-IrO_2_ formation, and has been realized in recent electrochemical experiments^10,12^. It has been argued that the residual presence of K^+^ ions in the nanoporous framework of amorphous iridium oxide (containing local structures of 1K + Ho-IrO_2_) might improve the overall catalytic performance and stability^10^.

For much higher applied potentials, the calculated stability region of IrO_3_ coincides well with previous studies^14^. Given that many non-equilibrium structures may be obtained by controlled synthesis conditions and a careful choice of reactants/precursors^10,24^, we are hopeful that more metastable iridium oxides (e.g. the amorphous phases and other nanoporous structures; see Supplementary Fig. 2) can be investigated for their (electro)chemical stability.

### Electronic structure

For the adsorption of the nucleophilic water molecule on the iridium oxide surface, the presence of an electrophilic oxygen atom may play a pivotal role as the susceptible adsorption site to bind the water molecule on the iridium oxide surface^4,15,18^. Here, we note that although the oxygen–oxygen bond formation does not necessarily involve the nucleophilic attack of a water molecule, following refs. ^15,35,36^, we collectively combine both the nucleophilic water attack and the oxygen–oxygen bond formation within the same mechanistic step (cf. Supplementary Eq. (16)). In past literature, there have been several discussions on how the so-called flexibility of the charge state of iridium center atom in the IrO_6_ octahedra (e.g. the varying ratio of Ir^3+^/Ir^4+^^10,18^) is correlated to the enhanced activity of iridium oxides for OER. However, a clear theoretical consensus is yet to be reached. Moreover, an apparent relationship between the flexible charge state of Ir and its structural motifs (e.g. in nanoporous and amorphous iridium oxides) is still lacking.

**Fig. 4:**
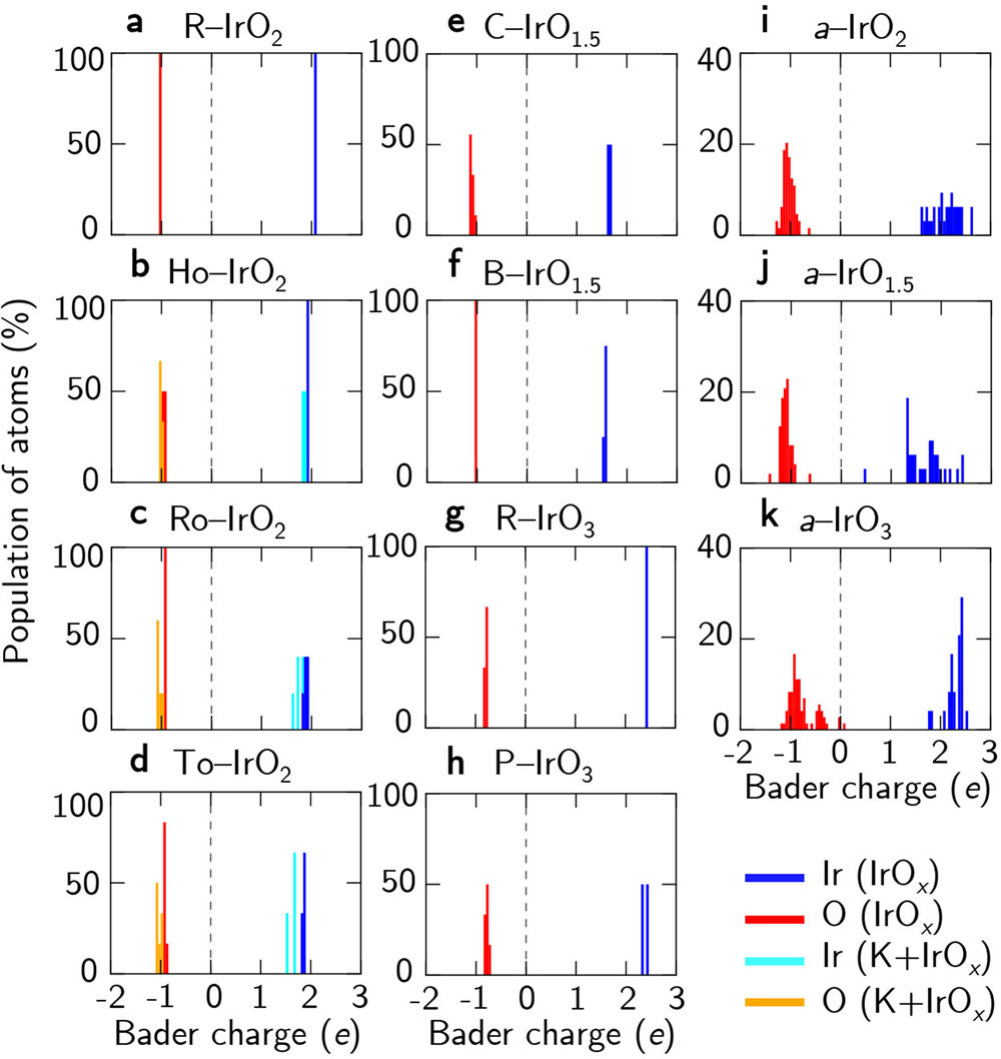
Bader charge population of both Ir and O atoms. **a** R-IrO_2_, **b** Ho-IrO_2,_ **c** Ro-IrO_2_, **d** To-IrO_2_, **e** C-IrO_1.5_, **f** B-IrO_1.5_, **g** R-IrO_3_, **h** P-IrO_3_. **i** *a*-IrO_2_. **j** *a*-IrO_1.5_, and **k** *a*-IrO_3_. The Bader charge histogram for Ir and O atoms (without K) are denoted in blue and red bars, while that for Ir and O atoms in K-intercalated nanoporous iridium oxides are shown in cyan and yellow bars, respectively.

To uncover the influence and relationship of the atomic charges of Ir and O atoms and the associated structural motifs, in Fig. 4, we first calculate and present the population histogram for the Bader charge of Ir and O atoms in the various iridium oxide systems. For R-IrO_2_, the Bader charge of the Ir atom is calculated as +2.06*e*, while that of crystalline IrO_1.5_ and IrO_3_ are determined to be between +1.52–+1.66 and +2.33–+2.42*e*, respectively. In the case of nanoporous IrO_2_ polymorphs, the Bader charges of Ir are calculated to be within the range of +1.81–+1.93*e*, which is somewhat less than that in R-IrO_2_ (+2.06*e*). Upon intercalation of K, the Bader charges of Ir (+1.52–+1.85*e*) are slightly reduced when compared to their pristine counterparts.

This reduction in the Bader charges of Ir can be correlated to the changes in the projected density-of-states (pDOS) for Ir in both the pristine and K-intercalated iridium oxides. In Supplementary Fig. 3, the Ir 5*d* states are calculated and plotted according to their Wyckoff positions. Noting the down-shift in the Ir 5*d* states to lower energy and the decreased Bader charges, it is indicative that electron transfer has occurred from the intercalated K atom to the nanoporous IrO_2_ host structure^12^.

Now, turning to the atomic charges of O atoms, we find a negative Bader charge value of −1.04*e* for R-IrO_2_, and the corresponding values for O atoms in crystalline IrO_1.5_, and IrO_3_ are between −1.14 to −1.02 and −0.85 to −0.74*e*, respectively. Here, we find that the oxygen atoms in the K-intercalated nanoporous iridium oxides become more nucleophilic (i.e. more negative in value) when compared to that of the pristine counterparts. For instance, the calculated Bader charges of O atoms in 1K + Ho-IrO_2_ are between −1.05 to −1.00*e*, as compared to that in pristine Ho-IrO_2_ (−0.99 to −0.94*e*). Referring to Fig. 4, similar trends are observed for 2K + Ro-IrO_2_ and 4K + To-IrO_2_. It now appears that K intercalation in these nanoporous iridium oxides adds extra thermodynamic stability but may not have a positive effect in generating more electrophilic oxygens needed for better OER performance.

Along the same vein of discussion, we notice a more flexible range of charge states for both the Ir and O atoms in the amorphous iridium oxides (namely, *a*-IrO_2_, *a*-IrO_1.5_, and *a*-IrO_3_). From Fig. 4i, the Bader charges of Ir and O atoms of *a*-IrO_2_ are calculated to be between +1.62–+2.61 and −1.29 to −0.62*e*, respectively (in contrast to +2.06 and −1.04*e* for R-IrO_2_). Similarly, the spread (hence, its flexibility) of the calculated Bader charges for *a*-IrO_1.5_ (Fig. 4j) and *a*-IrO_3_ (Fig. 4k) are found to range between +0.47–+2.40 (for Ir) and −1.44 to −0.64*e* (for O), and +1.75–+2.54 (for Ir) and −1.17 to +0.12*e* (for O), respectively.

It is worth noting that the very small positive values in the Bader charges of O atoms for *a*-IrO_3_ are attributed to oxygen clustering, as mentioned above. The variations in Bader charges of Ir atoms in amorphous iridium oxides are wider as compared to the crystalline and nanoporous analogs—indicating the possible presence of mixed valence states of Ir (i.e. Ir^3+^–Ir^6+^; see Supplementary Fig. 4) in these amorphous structures. It is clear that the diversity (or flexibility) found for the atomic charges can be attributed to the non-equivalent connectivity of the IrO_*x*_ polyhedra^10,37^, given more severe local structural distortions/disorders can be found in these amorphous iridium oxides. Thus, results from our DFT calculations now gravitate towards the same deduction and observation reported in recent experiments where the flexibility of the charge state of Ir (hence, the possibility of electrophilic O species) is determined as a key descriptor for enhanced OER performance in amorphous iridium oxides^18,19^.

**Fig. 5:**
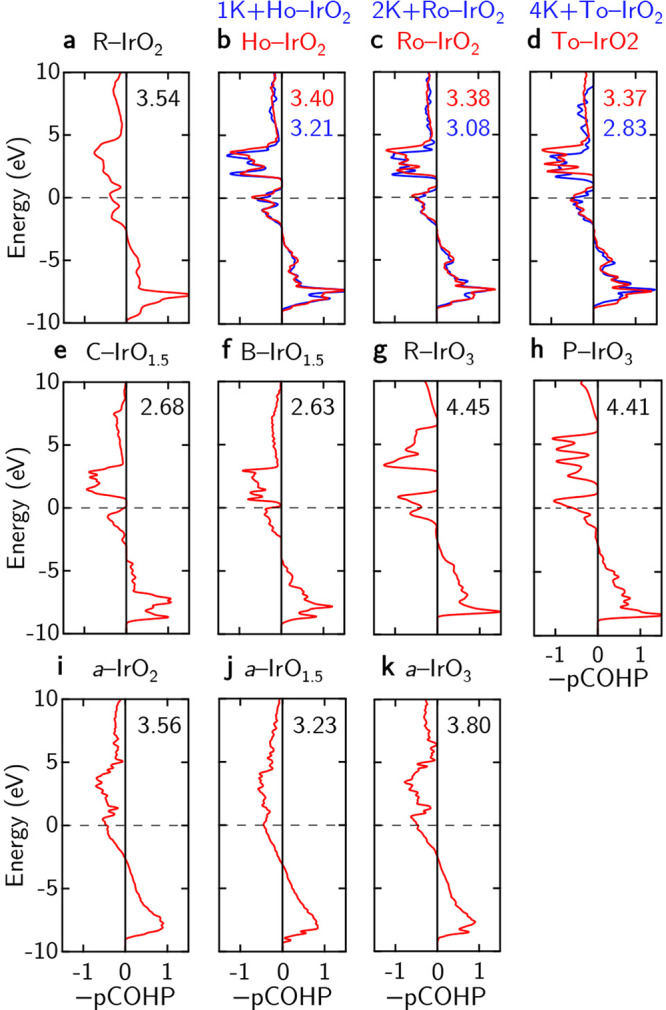
Averaged projected crystal orbital Hamilton population (−pCOHP) between the Ir and O atoms. **a** R-IrO_2_, **b** Ho-IrO_2_, **c** Ro-IrO_2_, **d** To-IrO_2_, **e** C-IrO_1.5_, **f** B-IrO_1.5_, **g** R-IrO_3_, **h** P-IrO_3_, **i** *a*-IrO_2_, **j** *a*-IrO_1.5_, and **k** *a*-IrO_3_. For each pCOHP plot, the (negative) integrated pCOHP (−IpCOHP) values (up to the Fermi level) is also indicated in the top-right hand corner. For the case of nanoporous IrO_2_, the −pCOHP curves and −IpCOHP values are denoted red and blue for the pristine and K-intercalated structures, accordingly. The Fermi level is set to zero in all pCOHP plots.

Besides the adsorption of nucleophilic water molecules on the surface of the OER catalysts, the desorption/evolution of the oxygen molecule is also a dominant step in the LOM scheme of OER^15^. To tie the electronic structure argument back to its chemical bonding characteristics, we calculate and report the averaged projected crystal orbital Hamilton population (pCOHP; cf. Supplementary Eq. (6)) between the Ir and O atom pairs in these oxides in Fig. 5. We note that it is by convention to report the negative of these values^38^. From the pCOHP, one can numerically discern the regions of bonding, anti-bonding, and non-bonding characteristics for a bond pair^39^. Thus, one can infer the bonding nature and strength of the Ir–O bond which will be important in aiding the understanding of the atomic processes of OER on IrO_*x*_ catalysts. For the iridium oxide structures considered in this work, the Ir–O bond length ranges from 1.5 to 2.5 Å.

From Fig. 5, the anti-bonding states of the Ir–O bond (corresponding to the negative values of the −pCOHP values) are found close to the Fermi-level for all iridium oxides. Upon K-intercalation for the nanoporous IrO_2_ structures, a down-shift to lower energies in the pCOHP plot is observed, corroborating well with the pDOS in Supplementary Fig. 3. To further aid our iono-covalent bonding character analysis^37,40,41^ in iridium oxides, we calculate the integrated pCOHP (IpCOHP) by integrating the pCOHP of specific bonding pairs of interest.

We observe that the magnitude of the −IpCOHP values for both the pristine and K-intercalated nanoporous IrO_2_ is reduced compared to that of R-IrO_2_, indicating a corresponding reduction in bond strength for the Ir–O bonds. Specifically, K-intercalation further reduces the −IpCOHP values, alluding that the intercalation of K^+^ ions in hollandite-type IrO_2_ is also experimentally shown to reduce the OER overpotential^42^. In the case of amorphous *a*-IrO_2_, *a*-IrO_1.5_, and *a*-IrO_3_ (in Fig. 5i, j, k, respectively), a similar magnitude of the −IpCOHP values is found as compared to that for R-IrO_2_, while that of crystalline IrO_1.5_ (in Fig. 5g, h) are determined to be higher. This trend seems to corroborate with the expected valence of Ir in these oxides, with the amorphous iridium oxides displaying an intermediate value due to the flexible charge states of Ir, as outlined above.

### Structure–property relationship and empirical regression model

**Fig. 6:**
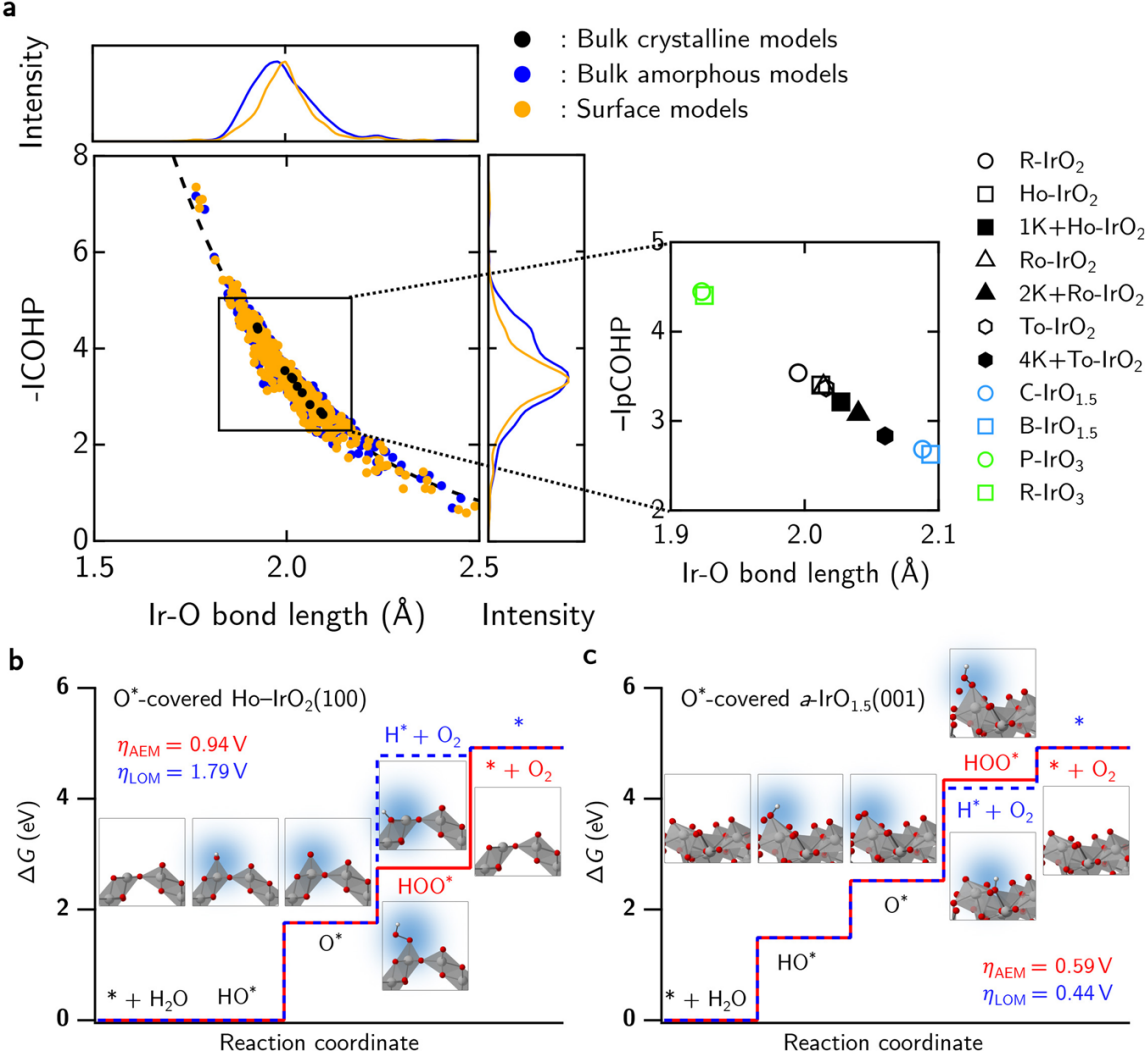
Chemical and reaction steps analysis for oxygen evolution reaction on iridium oxides. **a** Scatterplot for −IpCOHP versus Ir–O bond lengths for bulk crystalline and amorphous models and the surfaces of amorphous iridium oxides. The detailed plot for all crytalline/nanoporous iridium oxide phases are shown in the inset. The Δ*G* diagrams for OER via the adsorbate evolving mechanism (AEM; in red) and the lattice oxygen mechanism (LOM; in blue) for **b** O^*^-covered Ho-IrO_2_(100), and **c** O^*^-covered *a*-IrO_1.5_(001). The corresponding atomic structures for each reaction step are also provided alongside the Δ*G* diagrams. The iridium, oxygen, and hydrogen atoms are depicted as gray, red, and white spheres, respectively.

Now, to make sense of the electronic structure, chemical bonding, and atomic structure of these iridium oxides of various stoichiometries and polymorphic forms, we will now plot the specific −IpCOHP values versus the Ir–O bond length for each given iridium oxide, considering the varying stoichiometries and polymorphic forms in Fig. 6a. The nonlinear relationship between the −IpCOHP (i.e. the index for Ir–O bond strength) and Ir–O bond length nicely follows Pauling’s empirical relation on bond length versus bond strength^37,41^, indicating that a longer bond would typically result in a weaker bond in a nonlinear fashion.

More importantly, from Fig. 6a, this nonlinear behavior is well captured for the amorphous iridium oxides where a wider assortment of strong/weak Ir–O bonds (hence, shorter/longer Ir–O bonds) is found. Unlike the amorphous oxides of iridium, their crystalline and nanoporous counterparts show a highly linear behavior for a narrower range of bond lengths and bond strengths. This provides a rather intuitive picture as to why in previously reported experiments^10,18,19,21,43^, the amorphous forms of iridium oxide seem to outperform their crystalline analogs in OER. It is now evident that amorphous IrO_*x*_ of various stoichiometries can accommodate both strong and weak Ir–O bonds simultaneously to substantially provide sufficient thermodynamic stability and higher reactivity (e.g. in the case of OER), respectively. This then further lends support to the presence of flexible charge states of Ir (and thus, the ability to tailor a population of more electrophilic O species) in the amorphous iridium oxides, and explaining the enhancement in OER performance.

Extending this argument from the bulk phases of iridium oxides to their surfaces, we have chosen and constructed four different surface models—namely, R-IrO_2_(110), Ho-IrO_2_(100), *a*-IrO_2_(001), and *a*-IrO_1.5_(001)—and their calculated −IpCOHP values as a function of Ir–O bond length are shown in Fig. 6a. Once again, the Pauling-like behavior for the surface bonds in these iridium oxides holds true and thus affords an appealing descriptor to discuss the impact of flexible bonds on the OER—even on the surfaces of these oxides. To address the ongoing debate as to whether the AEM or the LOM mechanism occurs on these iridium oxide surfaces, we calculate the Gibbs energy, Δ*G* for the AEM and LOM mechanistic steps^36^ via the computational hydrogen electrode (CHE) approach^44^ while considering the effect of surface coverages under reaction conditions^45,46^. The Gibbs energy diagrams for O^*^-covered Ho-IrO_2_(100) and O^*^-covered *a*-IrO_1.5_(001) are presented in Fig. 6b, c, respectively. (The corresponding results for 2O${}_{{{{{{{{\rm{CUS}}}}}}}}}^{* }$-covered R-IrO_2_(110) and O^*^-covered *a*-IrO_2_(001) can be found in Supplementary Fig. 7a,  b. Further details are tabulated in Supplementary Tables 4 and 5).

Generally, for the crystalline phases of IrO_2_ (including the nanoporous IrO_2_ systems), the calculated value of *η*_LOM_ (~2 V) is almost two times larger than that of *η*_AEM_ (~1 V), as shown for O^*^-covered Ho-IrO_2_(100) in Fig. 6b and 2O${}_{{{{{{{{\rm{CUS}}}}}}}}}^{* }$-covered R-IrO_2_(110) in Supplementary Fig. 7a. This is inline with previous theoretical calculations^15,36^, and is in line with recent experiments^47^ where the contribution of the LOM mechanism to the OER on polycrystalline IrO_2_ is deemed negligible. In contrast, the calculated overpotential values for the amorphous (non)stoichiometric IrO_2_ phases differ less (e.g. the calculated *η*_AEM_ and *η*_LOM_ for O^*^-covered *a*-IrO_1.5_(001) are 0.59 and 0.44 V, respectively; see Fig. 6c) and are lower in magnitude when compared to their crystalline counterparts. In the same vein of discussion, we deduce that the smaller (and more similar) values of *η*_AEM_ and *η*_LOM_ for OER on the amorphous IrO_*x*_ surfaces may stem from their ability to display a more flexible range of Ir–O bond lengths (and hence bond strengths) as captured by the Pauling’s empirical relation in Fig. 6a.

To provide a perspective of how activated Ir–O bonds (i.e. stretched Ir–O bonds) may be correlated to the overall OER performance, we put forth a simple linear regression model to estimate the OER overpotential of these amorphous and nanoporous iridium oxides. From literature, we obtain the experimentally measured overpotential^12^ for R-IrO_2_ (0.40 V) and 1K + Ho-IrO_2_ (0.34 V) and correlate that to our DFT-calculated −IpCOHP for R-IrO_2_ (3.54) and 1K + Ho-IrO_2_ (3.21). From literature^12,48–51^, we have further collected the measured OER overpotentials for various oxides—namely, RuO_2_, SrIrO_3_, Sr_2_IrO_4_, Sr_4_IrO_6_, CuFeO_3_, SrFeO_3_, and CaFeO_3_—and performed −IpCOHP calculations using their DFT-optimized lattice parameters (where the initial lattice constants are taken from the Materials Project database^52^). Details of these values are tabulated in Table S3 of the Supplementary Information.

**Fig. 7:**
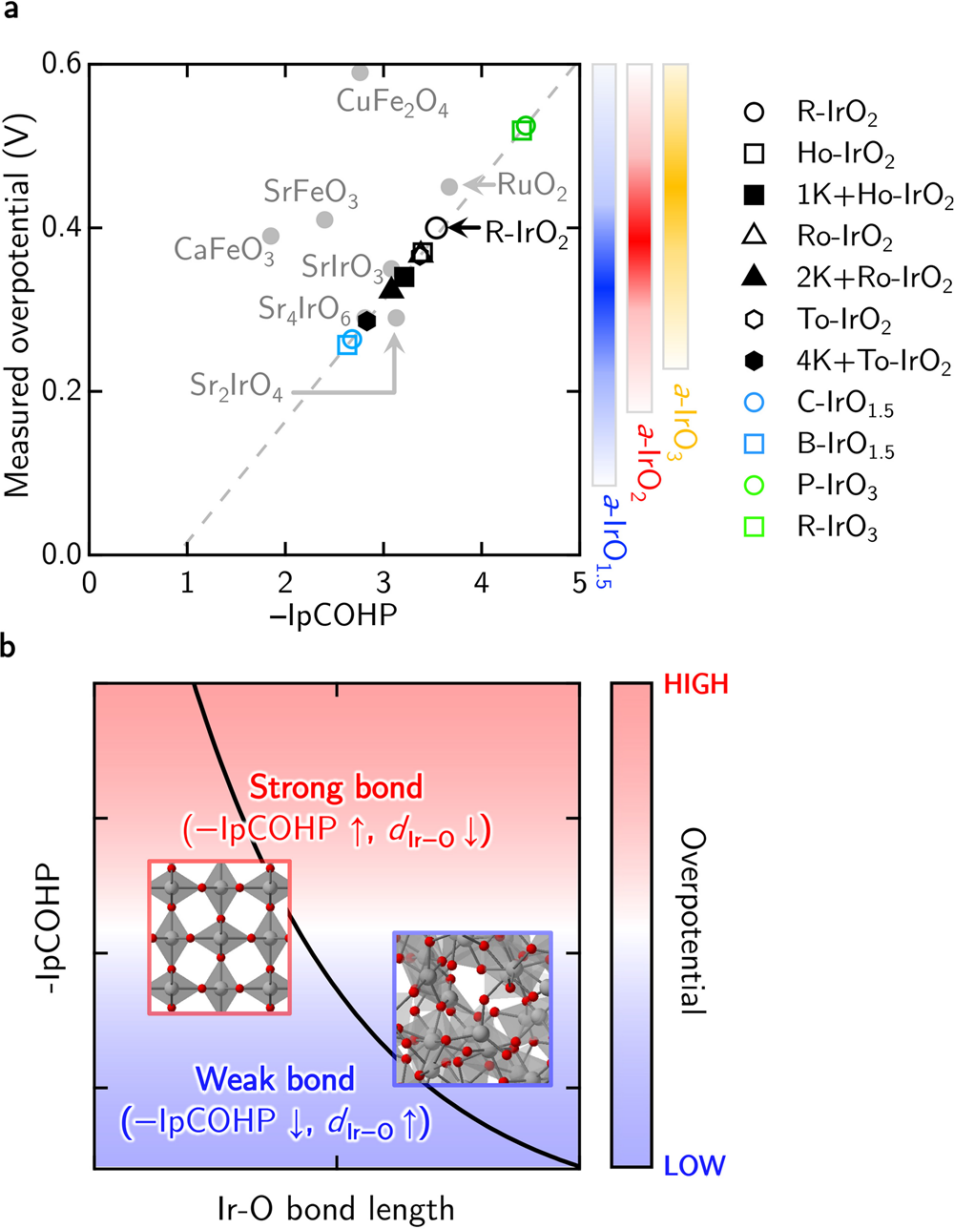
Empirical regression model for structure-property relations in various OER oxide catalysts. **a** The experimental overpotentials of RuO_2_, SrIrO_3_, Sr_2_IrO_4_, Sr_4_IrO_6_, CuFeO_3_, SrFeO_3_, and CaFeO_3_ are taken from refs. ^48–51^. The −IpCOHP values are thus calculated from the Ir–O, Ru–O, or Fe–O bonding, accordingly. The gray dotted line is a least-square linear regression using values determined for R-IrO_2_, 1K + Ho-IrO_2_, SrIrO_3_, Sr_2_IrO_4_, and Sr_4_IrO_6_, with the corresponding *R*^2^ value of 0.71. The range of overpotential values for the amorphous iridium oxides are provided from the linear regression model by considering the dispersion −IpCOHP plots shown in side-panel graph. **b** Schematic view of the empirical relationship between Ir–O bond length, −IpCOHP, and overpotential. The iridium and oxygen atoms are depicted as gray and red spheres, respectively, while the IrO_6_ octahedra is shaded in gray.

By using the experimentally measured overpotential values and our DFT-calculated −IpCOHP values of bulk R-IrO_2_, 1K + Ho-IrO_2_, SrIrO_3_, Sr_2_IrO_4_, and Sr_4_IrO_6_, a least-squared linear regression is plotted in Fig. 7a. In general, we find that RuO_2_ and the iridates (SrIrO_3_, Sr_2_IrO_4_, and Sr_4_IrO_6_) scatter close to the linear regression line while the ferrites (CuFeO_3_, SrFeO_3_, and CaFeO_3_) deviate from this linear behavior, highlighting our simple regression line may capture the needed chemistry to estimate the OER potentials of iridium oxides studied in this work. In this line of argument, RuO_2_ exhibits a larger overpotential due to stronger Ru–O bonds while the iridates may show comparable or even better OER performance as compared to the reference R-IrO_2_.

## Discussion

Using this empirical linear regression model (based on the Ir-based oxides), we can now empirically estimate the OER overpotential of the crystalline, nanoporous, and amorphous iridium oxides from their corresponding calculated −IpCOHP values. In particular, pristine nanoporous Ho-, Ro-, and To-IrO_2_ are predicted to exhibit a slightly lowered overpotential of 0.37, 0.37, and 0.36 V, respectively, in comparison to experimentally measured overpotential 0.4 V for R-IrO_2_^12^. Upon K-intercalation, a further decrease in overpotential values to 0.32 and 0.29 V is seen for 2K + Ro-IrO_2_ and 4K + To-IrO_2_, respectively. For crystalline IrO_1.5_, the estimated overpotential values are comparatively lower at 0.26 V while that for crystalline IrO_3_ is the largest at 0.52 V.

For the bulk amorphous iridium oxides, given a Pauling-like non-linear variation of −IpCOHP (in Fig. 6a), we take the statistical average (within the 90% confidence level) and plot the range of estimated overpotential values (as derived from the linear regression model) by considering the dispersion −IpCOHP plots shown in side-panel graph in Fig. 6a. This leads to a very similar estimated OER overpotential value of 0.4 V as compared to the reference R-IrO_2_. However, given the presence of flexible atomic charges and activated/stretched Ir–O bonds in these amorphous iridium oxides, a much lower limit of ~0.20 V may be attained (as estimated from the empirical linear regression model). This is qualitatively inline with our calculated overpotential trends when comparing the crystalline versus amorphous iridium oxide phases (Fig. 6b, c).

Combining this empirical regression model and the Pauling-like non-linear variation of −IpCOHP versus bond length (for both bulk and surfaces of iridium oxides), a schematic perspective plot (Fig. 7b) may be drawn to illustrate this empirical relationship between the bond length, bond strength, and OER overpotential values where flexible charges states and activated bonds may explain highly efficient Ir-based OER catalysts.

In conclusion, we have expanded and considered various nanoporous (with the inclusion of K-intercalation) and amorphous iridium oxides of different chemical stoichiometries. They are considered metastable with reference to the commonly reported ground state structures of IrO_2_ and IrO_3_. This report marks the first time amorphous oxides of iridium are discussed. Under an electrochemical environment, we predict that the intercalation of K in nanoporous IrO_2_ may be stable if the formation of rutile-type IrO_2_ is kinetically hindered. When considering the atomic Bader charges of these IrO_*x*_ polymorphs, it becomes apparent that nonequivalent connectivity in the amorphous structures strongly enhance the flexibility of the charge states of Ir, and hence promoting the presence of electrophilic oxygens in them, as compared to their crystalline counterparts. Using the COHP-bonding analysis, we obtain a Pauling-like relation between the Ir–O bond length versus bond strength for the Ir–O bonds in amorphous iridium oxides while a fairly linear trend is found for the crystalline analogs. This also corroborates with the proposal of flexible charges states and activated bonds for more efficient OER catalysis. Lastly, using an empirical regression model between the Ir–O bond characteristics and the measured OER overpotentials, we provide a perspective as to how these less understood metastable nanoporous and amorphous iridium oxides can offer a breakthrough in OER performance by a notable lowering of the anode overpotential. Here, we offer a fundamental atomistic picture to explain and reconcile the superior OER performance of sub-stoichiometric amorphous iridium oxides (where some channel-like microstructures have been observed) in recent OER experiments.

## Methods

The DFT calculations are performed employing the projector augmented wave (PAW) method^53^ as implemented in the Vienna Ab initio Simulation Package (VASP)^54,55^. The 6*s* and 5*d* states of Ir, 2*s* and 2*p* states of O, and 3*s*, 3*p*, and 4*s* states of K are explicitly considered as the valence states within the PAW approach. The optB86b exchange-correlation (*xc*) functional is used, treating the DFT *xc* energy using a self-consistent van der Waals-corrected semi-local generalized gradient approximation^56^. The optB86b *xc* functional has been shown to adequately describe various physicochemical properties of iridium oxides^57^. All DFT calculations have been tested for convergence of kinetic energy cutoff and **k**-points, where total energies and forces do not change more than 20 meV and 0.02 eV Å^−1^, respectively. A planewave kinetic energy cutoff of 500 eV and a Γ-centered **k**-point grid spacing of 0.15 Å^−1^ are used.

Ab initio molecular dynamics (*ai*MD) calculations are conducted at constant pressure (i.e. using the NPT ensemble) following the method of Parrinello and Rahman^58,59^. The Langevin thermostat is used to control the temperature modulation with a friction coefficient of 10 ps^−1^. To model the amorphous phases of iridium oxide with different stoichiometry, the 96-atom and 216-atom supercells of the cubic IrO_2_ pyrite phase, the 80-atom cell IrO_1.5_ bixbite phase, the 240-atom IrO_1.5_ corundum phase, and the 96-atom and 192-atom supercells of the $R\overline{3}c$ phase of IrO_3_ are adopted as initial atomic configurations for the respective stoichiometries. For these large supercell structures, the Brillouin zone is folded to the Γ-point and the kinetic cutoff energy is lowered to 300 eV. The initial structures are randomized at 3000 K for 10 ps (mimicking the melting process) and then quenched from 3000 to 100 K in 5 ps, with a cooling rate of 580K/ps. We further equilibrate the quenched structures at 300 K for another 10 ps in order to get more representative geometries at the temperature. For all *ai*MD calculations, a time step of 1 fs is used for the integration of the equations of motion.

The methods used to analyze the structural descriptors^25^, the (electrochemical) thermodynamic stability, and chemical bonding and orbital population via the LOBSTER code^38,60–63^ are detailed in the Supplementary Information (SI).

## Supplementary information

Supplementary Information

## Data Availability

The data that support the findings of this study are available within the article and its supplementary information. Specifically, atomic structures generated in this work have been deposited in Zenodo under accession code 10.5281/zenodo.5733499.
