# Supplementary material for: Activated chemical bonds in nanoporous and amorphous iridium oxides favor low overpotential for oxygen evolution reaction
Source: Nat Commun. 2022 Jun 8;13:3171. doi: 10.1038/s41467-022-30838-y (PMC9177587; doi:10.1038/s41467-022-30838-y)
Supplement: Supplementary file 1 — Supplementary Information [file 41467_2022_30838_MOESM1_ESM.pdf]

Supplementary Information

Activated Chemical Bonds In Nanoporous And Amorphous Iridium Oxides Favor Low Overpotential For Oxygen Evolution Reaction

S. Lee et al.

Supplementary Note 1. Methods

To analyze the detailed structural information quantitatively, the radial distribution function (RDF), $g(r)$, is calculated by

$$g(r) = \frac{dn}{4\pi r^2 dr \rho}; \quad \rho = \frac{N}{V} \quad , \tag{1}$$

where dn is defined as the number of atoms at a distance between r and $r + dr$, and N and V are the number of atoms and the volume of the periodic simulation cell, respectively.

In order to assess the energetic stability of the various polymorphs of IrO_2, $IrO_{1.5}$, IrO_3, and their K-intercalated phases, we calculate their formation energy (ΔH^{f}, in eV/atom) with respect to the bulk fcc phase of Ir and the O_2 molecule as follows,

$$\Delta H^{\mathrm{f}} = \frac{1}{N}\left(E_{\mathrm{K}_x\mathrm{IrO}_y} - \sum N_{\mathrm{M}} E_{\mathrm{M}}^{\mathrm{bulk}} - \frac{N_{\mathrm{O}}}{2} E_{\mathrm{O}_2}^{\mathrm{mlc}}\right) \quad , \tag{2}$$

where N, N_{M}, N_{O}, $E_{\mathrm{K}_x\mathrm{IrO}_y}$, $E_{\mathrm{M}}^{\mathrm{bulk}}$, and $E_{\mathrm{O}_2}^{\mathrm{mlc}}$ are the total number of atoms, the number of metal elements (M=Ir and K), and oxygen atoms, the total energy of bulk (K-intercalated) iridium oxide, the total energy of bulk metal (M=Ir and K; per atom) , and the total energy of the oxygen molecule, respectively. To estimate the vibrational entropic contribution, we have employed the Debye-Slater model (as implemented in the `Gibbs2` code [1]) to determine the vibrational energy, F^{vib}, as follows

$$F^{\mathrm{vib}} = \frac{9}{8} n k_{\mathrm{B}} \Theta_D + 3 n k_{\mathrm{B}} T \ln\left(1 - e^{-\Theta_D/T}\right) - n k_{\mathrm{B}} T D\left(\Theta_D/T\right) \quad , \tag{3}$$

where D is the Debye integral, Θ_D is the Debye temperature which is related to the Debye frequency, ω_D where $\Theta_D = \omega_D/k_{\mathrm{B}}$. Then the Gibbs energy of formation (ΔG^{f}) is calculated by scaling the vibrational entropic contribution at 300 and 600 K, notated with $\Delta G^{\mathrm{f}}(300\,\mathrm{K})$ and $\Delta G^{\mathrm{f}}(600\,\mathrm{K})$, respectively. Also we calculate the intercalation energy (ΔH^{int}, in eV/K atom) of K ion in the nanoporous IrO_2 structure as follows,

$$\Delta H^{\mathrm{int}} = \frac{1}{N_{\mathrm{K}}}\left(E_{\mathrm{K}_x\mathrm{IrO}_2} - E_{\mathrm{IrO}_2}^{\mathrm{frame}} - N_{\mathrm{K}} E_{\mathrm{K}}^{\mathrm{atom}}\right) \quad , \tag{4}$$

where N_{K}, $E_{\mathrm{IrO}_2}^{\mathrm{frame}}$, and $E_{\mathrm{K}}^{\mathrm{atom}}$ are the number of potassium atoms, the total energy of the

iridium oxide frame structure before K atoms are intercalated, and the total energy of the bulk potassium (per atom), respectively.

To investigate the relative electrochemical stabilities as a function of pH and electrode potential as known as the Pourbaix diagram, we calculate the reaction energy, $\Delta\mu$, corresponding the reaction path via atomic simulation environment (ASE) module [2]. The detailed reaction paths and corresponding equations for the reaction energy are notated on the Table S1 in the Supporting Information. For the chemical potential of the solid oxide compound, we use the Gibbs energy of formation at 300 K ($\Delta G^{\mathrm{f}}(300\,\mathrm{K})$). And to calculate the chemical potential of ions in solution, we express the chemical potential of ions in solution, μ_i, with the following equation

$$\mu_i = \mu_i^{\mathrm{exp.}} + RT \cdot \log([\mathrm{ion}]) \quad , \tag{5}$$

where $\mu_i^{\mathrm{exp.}}$, R, T and [ion] are the experimentally measured Gibbs energy of formation for the ion, the gas constant (8.314 J/mol K), temperature, and the concentration of the ion in solution, respectively. The experimental Gibbs energy of formation for IrO_4^- and K^+ ions are -2.04 and -2.93 eV/f.u., respectively [3, 4]. The ionic concentration for IrO_4^- and K^+ ions are set as 10^{-2} mol/L, mimicking the experimental synthesis condition of the Reference 5. The chemical potential of the water molecule, $\mu_{\mathrm{H_2O}}$, is set to -2.46 eV, following the previous systematic studies [6, 7].

The projected crystal orbital Hamilton population (pCOHP) is calculated using the LOBSTER code [8–12] for chemical bonding analysis as

$$\mathrm{pCOHP}_{\mu v}(E, \mathbf{k}) = \sum \mathcal{R}\left[P_{\mu vj}^{(\mathrm{proj})}(\mathbf{k}) H_{v\mu}^{(\mathrm{proj})}(\mathbf{k})\right] \times \delta\left(\epsilon_j(\mathbf{k}) - E\right) \quad , \tag{6}$$

where $P_{\mu vj}^{(\mathrm{proj})}(\mathbf{k})$ and $H_{v\mu}^{(\mathrm{proj})}(\mathbf{k})$ are projected density matrix and projected Hamilonian matrix elements for every band j and every $\mathbf{k}$-point, respectively. The delta function (δ) expresses that the density matrix only has a nonzero value at the specific band energy $\epsilon_j(\mathbf{k})$. Finally, to examine the charge state of the each atoms in the atomic structure, we calculate the Bader charges using the optB86b *xc* functional [13].

To model surfaces of iridium oxides, we consider the supercell slab models of R-IrO_2(110) $p(2 \times 3)$, Ho-IrO_2(100) $p(3 \times 1)$, a-IrO_2(001) $p(1 \times 1)$, and a-$IrO_{1.5}$(001) $p(1 \times 1)$, ensuring a

lateral distance of $\sim 9\,\text{Å}$ betweeen the molecule adsorption and their periodic images. The surface slab models for amorphous iridium oxide are generated from the (001) plane of our optimized bulk structures of a-IrO_2 and a-$IrO_{1.5}$. All surfaces are constructed using asymmetric periodic slab models with at least $20\,\text{Å}$ of vacuum height and a dipole correction has been applied to avoid unphysical interactions between neighboring slabs in the z-direction. And the bottom-most iridium and oxygen atoms ($\sim 4.5\,\text{Å}$ in thickness) are fixed at their bulk positions, while the other atoms are fully relaxed. For consistency with the bulk calculations, all structures have been optimized using the optB86b xc functional [14], and a Γ-centered $\mathbf{k}$-point grid spacing of $0.15\,\text{Å}^{-1}$ is used. This corresponds to a $\mathbf{k}$-point grid of $4 \times 7 \times 1$, $7 \times 3 \times 1$, and $1 \times 1 \times 1$ for R-IrO_2(110), Ho-IrO_2(100) and amorphous surface slab models, respectively.

To consider surface coverage, we calculate relative Gibbs energy following the previous theoretical studies [15–17]. We benchmark and present the calculated Pourbaix diagram of R-IrO_2(110) in Fig. S6 following the surface naming conventions in Reference 17, and applied the same method to Ho-IrO_2(100), a-IrO_2, and a-$IrO_{1.5}$. For Ho-IrO_2(100) phases, we adsorbed O (O^*-covered) or OH (OH^*-covered) species on all coordinately unsaturated sites (CUS). For amorphous structures (a-IrO_2(001) and a-$IrO_{1.5}$(001)), we added O (O^*-covered) or OH (OH^*-covered) species each for the outmost five iridium atoms. Further details are tabulated in Table S4.

To calculate the theoretical OER overpotential, we have employed the computational hydrogen electrode (CHE) approach due to Nørskov et al. [18]. The change of Gibbs energy (ΔG) are calculated using

$$\Delta G = \Delta E + \Delta \text{ZPE} + \int C_p \, dT - T\Delta S \quad , \tag{7}$$

where ΔE, ΔZPE, C_p, T, ΔS are the total energy calculated from DFT, the change of zero-point energy of gas-phase species upon adsorption, the heat capacity of gas-phase species without adsorption, temperature, and the entropy, respectively. Following the previous literature, the zero point energy of the absorbed species are calculated on the Ho-IrO_2(100) surface and it is adapted for other surfaces as well [19]. To calculate the zero-point energy of molecules and adsorbed species, the vibrational frequencies of the adsorbed species are determined within the harmonic approximation model where the contributions of the slab

to the vibrational energy are not adapted [20]. Also, the enthalpic temperature correction term ($\int C_p\, dT$) for the adsorbate/substrate system is assumed to be negligible but has been included for gas-phase molecule calculations only following the previous report [21]. The entropy values of the molecular species are also collected from the previous investigation [22]. We used the standard conditions so the temperature is taken as 298.15 K.

More specifically, we have considered the AEM mechanistic path as follows:

$$\mathrm{H_2O} + {}^* \rightarrow \mathrm{HO}^* + \mathrm{H}^+ + e^- \tag{8}$$

$$\mathrm{HO}^* \rightarrow \mathrm{O}^* + \mathrm{H}^+ + e^- \tag{9}$$

$$\mathrm{O}^* + \mathrm{H_2O} \rightarrow \mathrm{HOO}^* + \mathrm{H}^+ + e^- \tag{10}$$

$$\mathrm{HOO}^* \rightarrow^* + \mathrm{O_2} + \mathrm{H}^+ + e^- \tag{11}$$

The change of the Gibbs energy for the reaction path following Equations 8 to 11 can be expressed as Equation 12 to 15 respectively,

$$\Delta G_1 = E_{\mathrm{DFT}}^{\mathrm{HO}^*} - E_{\mathrm{DFT}}^{*} - \left(E_{\mathrm{DFT}}^{\mathrm{H_2O(g)}} - 1/2E_{\mathrm{DFT}}^{\mathrm{H_2(g)}}\right) + \left(\Delta\mathrm{ZPE} - T\Delta S^0\right) - eU \quad , \tag{12}$$

$$\Delta G_2 = E_{\mathrm{DFT}}^{\mathrm{O}^*} - E_{\mathrm{DFT}}^{\mathrm{HO}^*} + 1/2E_{\mathrm{DFT}}^{\mathrm{H_2(g)}} + \left(\Delta\mathrm{ZPE} - TS^0\right) - eU \quad , \tag{13}$$

$$\Delta G_3 = E_{\mathrm{DFT}}^{\mathrm{HOO}^*} - E_{\mathrm{DFT}}^{\mathrm{O}^*} - \left(E_{\mathrm{DFT}}^{\mathrm{H_2O(g)}} - 1/2E_{\mathrm{DFT}}^{\mathrm{H_2(g)}}\right) + \left(\Delta\mathrm{ZPE} - T\Delta S^0\right) - eU \quad , \tag{14}$$

$$\Delta G_4 = E_{\mathrm{DFT}}^{*} - E_{\mathrm{DFT}}^{\mathrm{HOO}^*} + \left(2E_{\mathrm{DFT}}^{\mathrm{H_2O(g)}} - 3/2E_{\mathrm{DFT}}^{\mathrm{H_2(g)}}\right) + 4.92 + \left(\Delta\mathrm{ZPE} - T\Delta S^0\right) - eU \quad , \tag{15}$$

where E^{*}_{DFT}, $E^{\mathrm{HO}^*}_{\mathrm{DFT}}$, $E^{\mathrm{O}^*}_{\mathrm{DFT}}$, $E^{\mathrm{HOO}^*}_{\mathrm{DFT}}$,$E^{\mathrm{H_2O(g)}}_{\mathrm{DFT}}$, $E^{\mathrm{H_2(g)}}_{\mathrm{DFT}}$, and U represent the DFT total energy of clean surface slab of iridium oxide, HO adsorbed surface slab, O adsorbed surface slab, HOO adsorbed surface slab, H_2O molecule and H_2 molecule, and electrode potential, respectively.

In the case of the LOM mechanistic pathway, following Reference 19, the first two mechanistic steps are taken as the same with the AEM (Equations 8 and 9) while the last two mechanistic steps are expressed using Equations 16 and 17:

$$\mathrm{O}^* + \mathrm{O}_{lat} + \mathrm{H_2O} \rightarrow \mathrm{H}^* + \mathrm{O}_{lat} + \mathrm{O_2} + \mathrm{H}^+ + e^- \tag{16}$$

$$\mathrm{H}^* + \rightarrow^* + \mathrm{H}^+ + e^- \tag{17}$$

The change in Gibbs energy for Equations 16 and 17 are given by Equations 18 and 19, respectively.

$$\Delta G_3 = E^{\mathrm{H}^*}_{\mathrm{DFT}} - E^{\mathrm{O}^*}_{\mathrm{DFT}} + \left(E^{\mathrm{H_2O(g)}}_{\mathrm{DFT}} - 3/2E^{\mathrm{H_2(g)}}_{\mathrm{DFT}}\right) + 4.92 + \left(\Delta\mathrm{ZPE} - T\Delta S^0\right) - eU \quad , \tag{18}$$

$$\Delta G_4 = E^{*}_{\mathrm{DFT}} - E^{\mathrm{H}^*}_{\mathrm{DFT}} + 1/2E^{\mathrm{H_2(g)}}_{\mathrm{DFT}} + \left(\Delta\mathrm{ZPE} - T\Delta S^0\right) - eU \quad , \tag{19}$$

where $E^{\mathrm{H}^*}_{\mathrm{DFT}}$ represents the DFT total energy of the H atom adsorbed on the surface of iridium oxide.

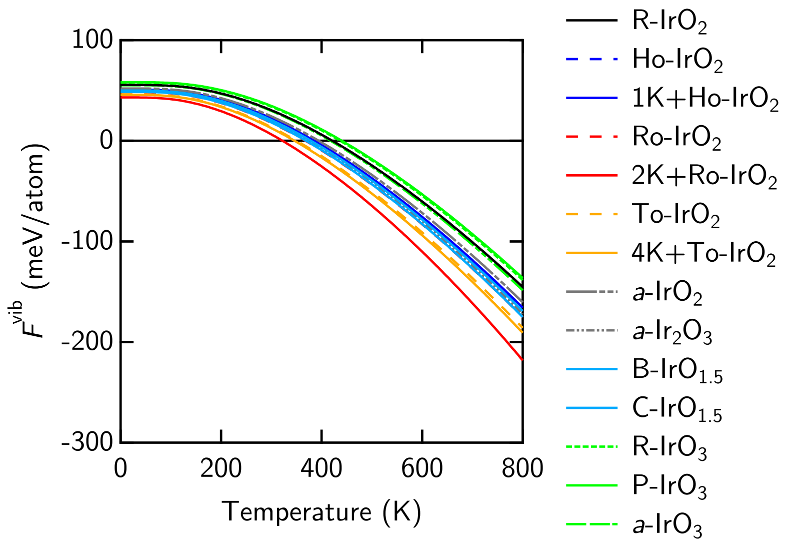

Supplementary Fig. 1. Vibrational energy, F^{vib}, of the various iridium oxide structures as a funtion of temperature calculated via Equation 4 using the optB86b xc functional.

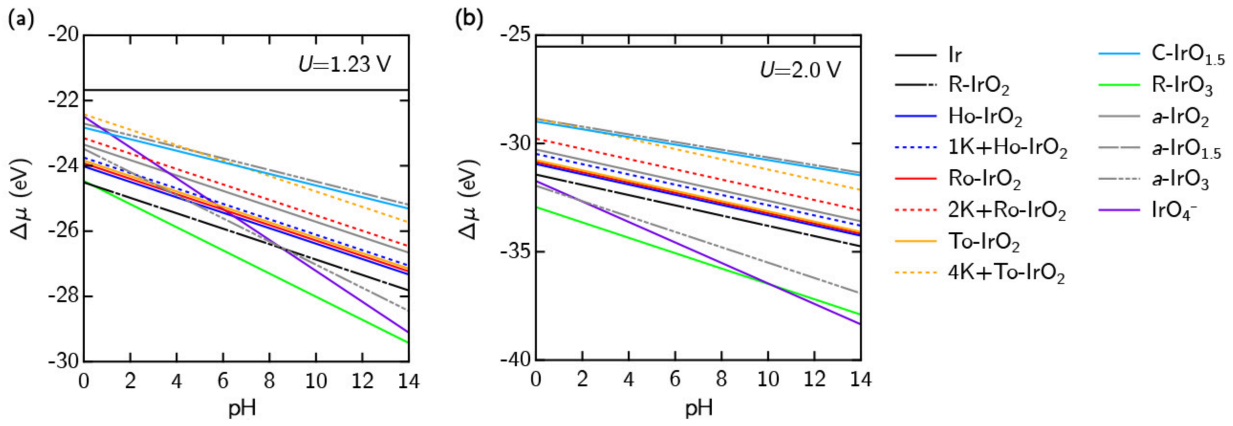

Supplementary Fig. 2. Change in chemical potential ($\Delta\mu$) for various iridium oxides plotted as a function of pH at different electrode potentials of (a) $U = 1.23\,\mathrm{V}$ and (b) $U = 2.0\,\mathrm{V}$, using the Equation S5 and Table S1.

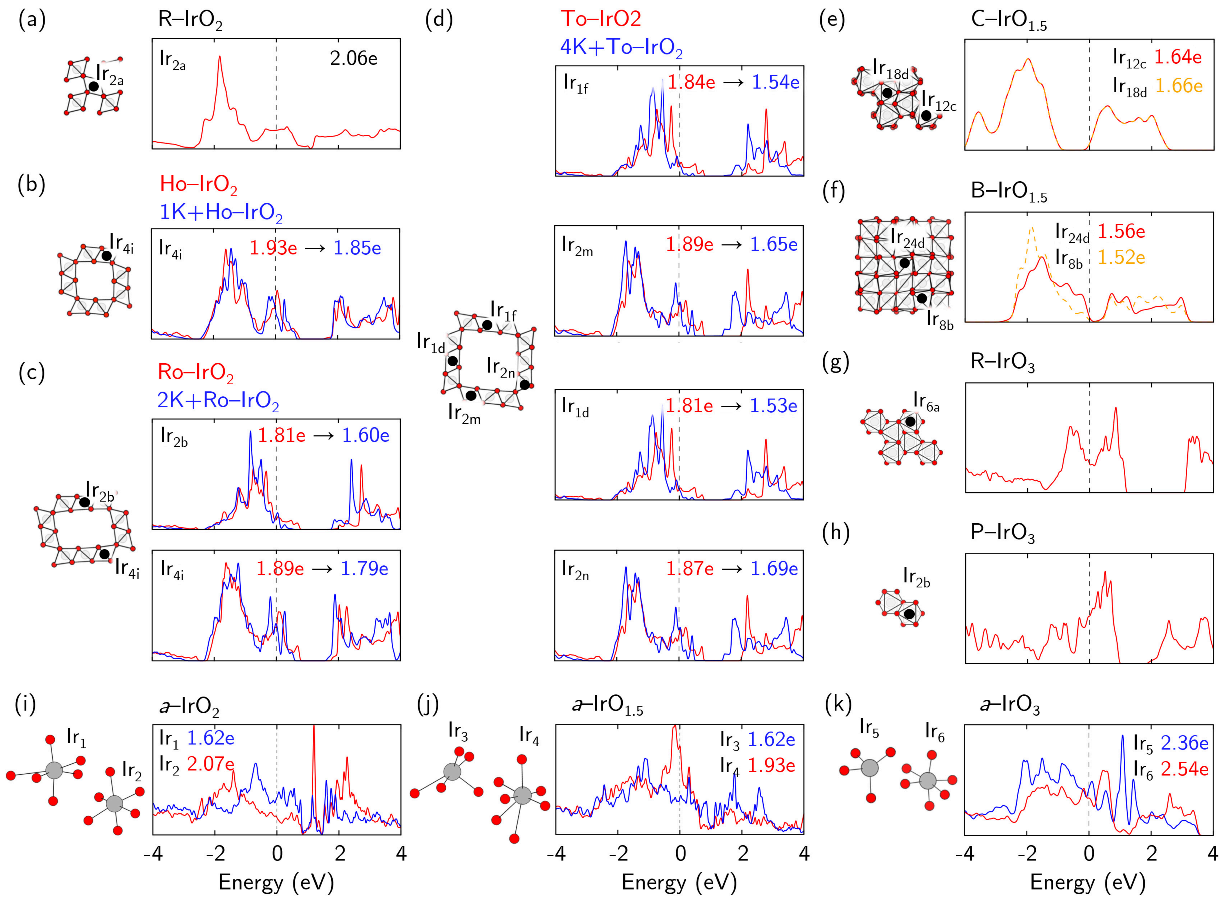

Supplementary Fig. 3. The partial density-of-states (pDOS) for 5*d* orbital and the bader charge of the specific Ir atom specified in the crystal structure of IrO_2 of (a) rutile (R-IrO_2), (b) hollandite (Ho-IrO_2), (c) romanechite (Ro-IrO_2), and (d) todorokite (To-IrO_2), and $IrO_{1.5}$ of (e) corundum (C-$IrO_{1.5}$), and (f) bixbite (B-$IrO_{1.5}$) structure, and IrO_3 of (g) R-IrO_3, and (h) P-IrO_3 structures, and amorphous phases of (i) IrO_2, (j) $IrO_{1.5}$, and (k) IrO_3, respectively. On the pDOS plot in (b)-(d), the red and blue line is depicted as the 5*d* orbital before and after the K atom is intercalated. The specific Ir atoms we picked are notated with the Wyckoff postion.

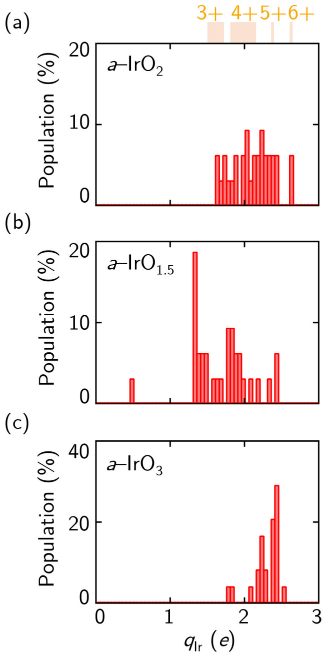

Supplementary Fig. 4. The Bader charge histogram for Ir atoms in (a) a-IrO_2, (b) $IrO_{1.5}$, and (c) IrO_3. The Bader charge for Ir^{3+}, Ir^{4+}, Ir^{5+}, and Ir^{6+} are calculated from the crystalline $IrO_{1.5}$, IrO_2, $IrO_{2.5}$, and IrO_3 phases, respectively.

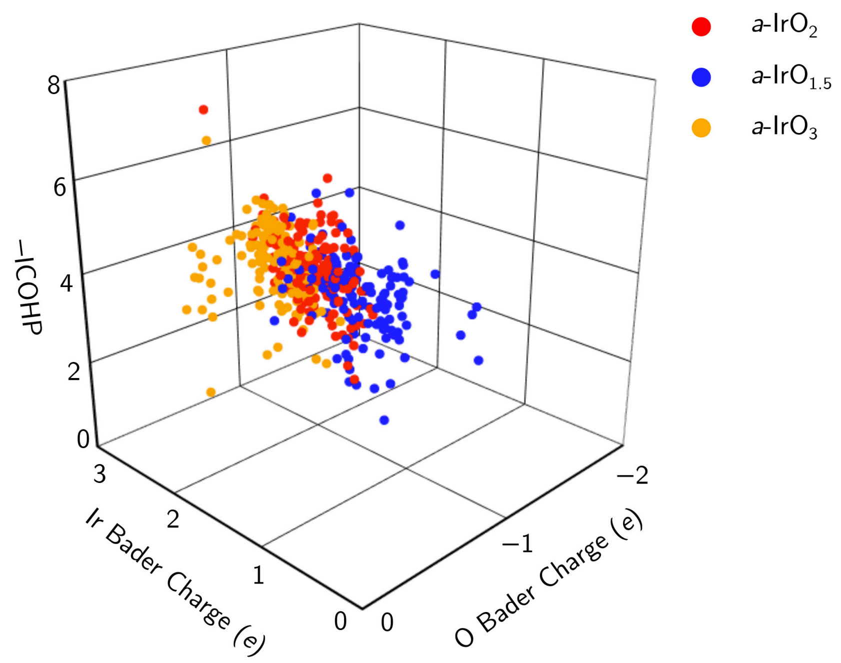

Supplementary Fig. 5. The calculated −IpCOHP as a function of the Bader charges of the iridium and oxygen atoms in the atomic structures of *a*-IrO_2, $IrO_{1.5}$, and IrO_3. The red, blue, and orange circular markers are to denote the *a*-IrO_2, $IrO_{1.5}$, and IrO_3, respectively.

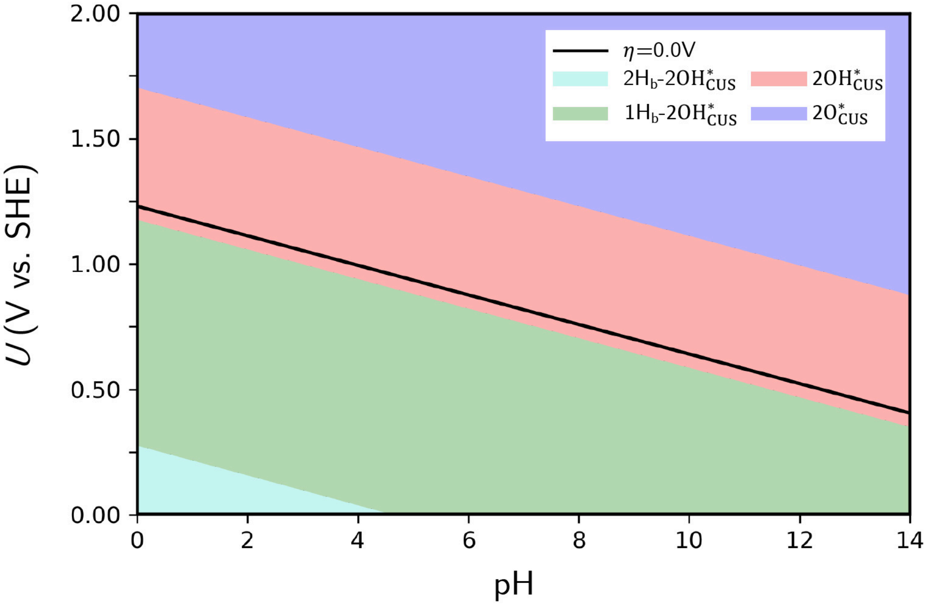

Supplementary Fig. 6. Calculated Pourbaix diagram for the R-IrO_2(110) surfaces. O* and OH* mean CUS are fully covered respectively and 1H_b-OH* and 1H_b-OH* mean half of bridge site are covered by H and all of bridge site are covered by H, respectively. Also, black line means theoretical minimum potential for water splitting.

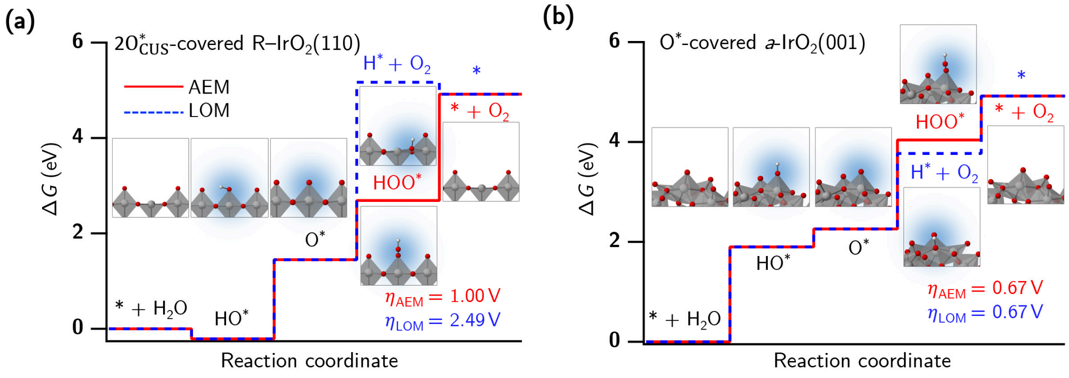

Supplementary Fig. 7. The Gibbs energy (ΔG) diagrams for OER via the adsorbate evolving mechanism (AEM) and the lattice oxygen mechanism (LOM) for (a) $2O^*_{CUS}$-covered R-IrO_2(110), and (b) O^*-covered a-IrO_2(001). The Gibbs energy diagrams for the AEM and LOM are denoted by red and blue lines, respectively. The corresponding atomic structures for each reaction step are also provided alongside the Gibbs energy diagrams. The iridium, oxygen, and hydrogen atoms are depicted as gray, red, and white spheres, respectively, while the IrO_6 octahedra is shaded in gray. The calculated overpotential for AEM (η_{AEM}) and LOM (η_{LOM}) are shown in each plot.

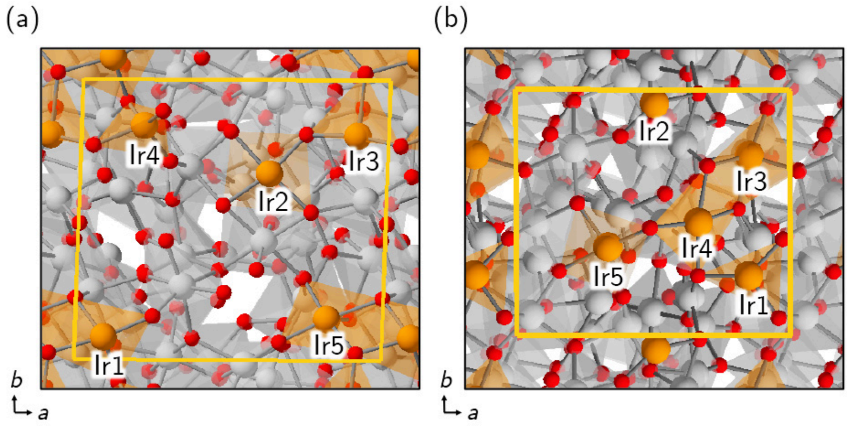

Supplementary Fig. 8. Top view of the atomic structures for the (a) a-IrO_2(001) and (b) a-$IrO_{1.5}$(001) surfaces. The sites chosen to calculate the overpotentials listed in Table S5 are labelled accordingly in the figure. The surface unit cell is represented by the lines in yellow.

Supplementary Table I. Chemical equations for the change in chemial potential ($\Delta\mu$ in eV), where μ(Ir) and μ(K) are taken as the references for the chemical potentials at standard conditions, U_p the electrode potential in V, e the charge of the electron, k_B the Boltzmann constant, and T the ambient temperature.

Reaction path	$\Delta\mu$
$\mathrm{Ir} + 2\mathrm{H_2O} \rightarrow$	$\Delta\mu(\mathrm{IrO_2} - \mathrm{Ir}) = \mu(\mathrm{IrO_2}) - \mu(\mathrm{Ir}) - 2\mu_{\mathrm{H_2O}} - 4eU_\mathrm{p}$
$\mathrm{IrO_2} + 4\mathrm{H^+} + 4\mathrm{e^-}$	$- 4k_\mathrm{B}T\ln(10)\mathrm{pH}$
$x\mathrm{K} + \mathrm{Ir} + 2\mathrm{H_2O} \rightarrow$	$\Delta\mu(\mathrm{K}_x\mathrm{IrO_2} - \mathrm{Ir} - x\mathrm{K}) = \mu(\mathrm{K}_x\mathrm{IrO_2}) - \mu(\mathrm{Ir}) - x\mu(\mathrm{K}) - 2\mu_{\mathrm{H_2O}}$
$\mathrm{K}_x\mathrm{IrO_2} + 4\mathrm{H^+} + (4{+}x)\mathrm{e^-}$	$- (4{+}x)eU_\mathrm{p} - 4k_\mathrm{B}T\ln(10)\mathrm{pH}$
$\mathrm{Ir} + 1.5\mathrm{H_2O} \rightarrow$	$\Delta\mu(\mathrm{IrO_{1.5}} - \mathrm{Ir}) = \mu(\mathrm{IrO_{1.5}}) - \mu(\mathrm{Ir}) - 1.5\mu_{\mathrm{H_2O}}$
$\mathrm{IrO_{1.5}} + 3\mathrm{H^+} + 3\mathrm{e^-}$	$- 3eU_\mathrm{p} - 3k_\mathrm{B}T\ln(10)\mathrm{pH}$
$\mathrm{Ir} + 3\mathrm{H_2O} \rightarrow$	$\Delta\mu(\mathrm{IrO_3} - \mathrm{Ir}) = \mu(\mathrm{IrO_3}) - \mu(\mathrm{Ir}) - 3\mu_{\mathrm{H_2O}}$
$\mathrm{IrO_3} + 6\mathrm{H^+} + 6\mathrm{e^-}$	$- 6eU_\mathrm{p} - 6k_\mathrm{B}T\ln(10)\mathrm{pH}$
$\mathrm{Ir} + 4\mathrm{H_2O} \rightarrow$	$\Delta\mu(\mathrm{IrO_4}^- - \mathrm{Ir}) = \mu(\mathrm{IrO_4}^-) - \mu(\mathrm{Ir}) - 4\mu_{\mathrm{H_2O}} - 7eU_\mathrm{p}$
$\mathrm{IrO_4}^- + 8\mathrm{H^+} + 7\mathrm{e^-}$	$- 8k_\mathrm{B}T\ln(10)\mathrm{pH}$

Supplementary Table II. The limits of the domains and the corresponding equilibrium formulas, where U_e and pH are the equilibrium electrode potential in V and the pH values, respectively.

Limits of the domains	Equilibrium formula
Ir/R-IrO_2	$U_e = 0.522 - 0.059$ pH
Ir/Ho-IrO_2	$U_e = 0.645 - 0.059$ pH
R-IrO_2/IrO_3	$U_e = 1.252 - 0.059$ pH
Ho-IrO_2/IrO_3	$U_e = 1.008 - 0.059$ pH
IrO_3/IrO_4^-	$U_e = 3.201 - 0.118$ pH
Ho-IrO_2/1K+Ho-IrO_2	$U_e = 0.168$
Ir/1K+Ho-IrO_2	$U_e = 0.677 - 0.063$ pH

Supplementary Table III. The experimental overpotential and electrolyte of various oxides for OER catalyst. A reversible hydrogen electrode (RHE) is set for a reference electrode. The specific measurement conditions for OER overpotential are also clarified in brackets. We perform −IpCOHP calculations using their DFT-optimized lattice parameters (where the initial lattice constants are taken from the Materials Project database [23]). All calculations are done with the optB86b *xc* functional.

MP-ID	Catalyst	−IpCOHP	Overpotential (V)	Current density (mA/cm^2)	Electrolyte	Ref.
mp-19115	$CaFeO_3$	1.85	0.39	0.5	0.1 M KOH	24
mp-510624	$SrFeO_3$	2.40	0.41	0.5	0.1 M KOH	24
mp-770107	$CuFe_2O_4$	2.76	0.59	0.5	0.1 M KOH	24
mp-1193907	$SrIrO_3$	3.08	0.35	10	0.1 M $HClO_4$	25
mp-4998	Sr_2IrO_4	3.13	0.29	10	0.1 M $HClO_4$	25
mp-9039	Sr_4IrO_6	2.81	0.29	10	0.1 M $HClO_4$	25
-	R-IrO_2	3.54	0.40	10	0.1 M $HClO_4$	26
-	1K+Ho-IrO_2	3.21	0.34	10	0.1 M $HClO_4$	26
mp-825	RuO_2	3.67	0.45	10	0.1 M $HClO_4$	27

Supplementary Table IV. Relative Gibbs energy (ΔG) and relative Gibbs energy at pH=0, U_{SHE} 1.68 V for $2O^{*}_{\mathrm{CUS}}$-, $2OH^{*}_{\mathrm{CUS}}$-, $1H_{\mathrm{b}}$-OH^{*}_{CUS}-, and $2H_{\mathrm{b}}$-OH^{*}_{CUS}-covered R-IrO_2(110), and O^{*}-, and OH^{*}-covered Ho-IrO_2(100), *a*-IrO_2(001), and *a*-$IrO_{1.5}$(001) where U_{p} the electrode potential in V, e the charge of the electron, k_{B} the Boltzmann constant, and T the ambient temperature..

Structure	Coverage species	Relative Gibbs energy ΔG (eV)	Relative Gibbs energy (eV) at pH=0, U_{SHE} 1.68 V
R-IrO_2(110)	$2O_{\mathrm{CUS}}$	$1.24 - 2eU_{\mathrm{p}} - 2k_{\mathrm{B}}T\ln(10)\mathrm{pH}$	−2.12
	$2OH_{\mathrm{CUS}}$	$-0.45 - eU_{\mathrm{p}} - k_{\mathrm{B}}T\ln(10)\mathrm{pH}$	−2.13
	$1H_{\mathrm{b}}$-$2OH_{\mathrm{CUS}}$	$-1.04 - 0.5eU_{\mathrm{p}} - 0.5k_{\mathrm{B}}T\ln(10)\mathrm{pH}$	−1.88
	$2H_{\mathrm{b}}$-$2OH_{\mathrm{CUS}}$	−1.18	−1.18
Ho-IrO_2(100)	O	$1.41 - 2eU_{\mathrm{p}} - 2k_{\mathrm{B}}T\ln(10)\mathrm{pH}$	−1.95
	OH	$0.14 - eU_{\mathrm{p}} - k_{\mathrm{B}}T\ln(10)\mathrm{pH}$	−1.54
a-IrO_2(001)	O	$1.72 - 2eU_{\mathrm{p}} - 2k_{\mathrm{B}}T\ln(10)\mathrm{pH}$	−1.64
	OH	$0.59 - eU_{\mathrm{p}} - k_{\mathrm{B}}T\ln(10)\mathrm{pH}$	−1.09
a-$IrO_{1.5}$(001)	O	$1.61 - 2eU_{\mathrm{p}} - 2k_{\mathrm{B}}T\ln(10)\mathrm{pH}$	−1.75
	OH	$0.58 - eU_{\mathrm{p}} - k_{\mathrm{B}}T\ln(10)\mathrm{pH}$	−1.10

Supplementary Table V. The change in Gibbs energy (ΔG_i) for each oxygen evolution reaction (OER) mechanistic path (where $i = 1, 2, 3$, and 4), via the adsorbate evolving mechanism (AEM) and the lattice oxygen mechanism (LOM), and the overpotentials (η in V) for $2O^*_{CUS}$- and $2OH^*_{CUS}$-covered R-IrO_2(110), O^*-covered Ho-IrO_2(100), O^*-covered a-IrO_2(001), and O^*-covered a-$IrO_{1.5}$(001). In case of the amorphous oxide surfaces, the labels for the specific reaction sites are shown in Fig. S8.

Structure	Site	Mechanism	ΔG_1 (eV)	ΔG_2 (eV)	ΔG_3 (eV)	ΔG_4 (eV)	η (V)
$2O^*_{CUS}$-covered R-IrO_2(110)	1	AEM	−0.21	1.66	1.24	2.23	1.00
		LOM			3.72	−0.25	2.49
$2OH^*_{CUS}$-covered R-IrO_2(110)	1	AEM	−0.49	1.70	1.38	2.34	1.11
		LOM			3.86	−0.15	2.63
O^*-covered Ho-IrO_2(100)	1	AEM	0.00	1.76	0.99	2.17	0.94
		LOM			3.02	0.14	1.79
O^*-covered a-IrO_2(001)	1	AEM	0.47	1.07	1.48	1.90	0.67
		LOM			3.08	0.30	1.85
	2	AEM	0.73	0.93	2.13	1.14	0.90
		LOM			2.52	0.75	1.29
	3	AEM	0.29	1.34	1.35	1.94	0.71
		LOM			2.80	0.49	1.57
	4	AEM	1.90	0.36	1.78	0.88	0.67
		LOM			1.51	1.15	0.67
	5	AEM	0.49	1.45	1.49	1.49	0.26
		LOM			2.17	0.81	0.94
O^*-covered a-$IrO_{1.5}$(001)	1	AEM	1.49	1.03	1.82	0.58	0.59
		LOM			1.67	0.74	0.44
	2	AEM	−0.60	1.17	1.39	2.95	1.72
		LOM			2.95	1.39	1.72
	3	AEM	0.42	1.15	1.61	1.74	0.51
		LOM			2.76	0.58	1.53
	4	AEM	0.95	0.75	2.37	0.85	1.14
		LOM			2.49	0.73	1.26
	5	AEM	0.93	1.88	1.02	1.08	0.65
		LOM			1.45	0.65	0.65

SUPPLEMENTARY REFERENCES

[1] A. Otero-de-la Roza, D. Abbasi-Pérez, V. Luaña. Gibbs2: A New Version of the Quasiharmonic Model Code. II. Models for Solid-state Thermodynamics, Features and Implementation. *Comput. Phys. Commun.* **2011**, *182*, 2232.

[2] A. H. Larsen, J. J. Mortensen, J. Blomqvist, I. E. Castelli, R. Christensen, M. Dułak, J. Friis, M. N. Groves, B. Hammer, C. Hargus, E. D. Hermes, P. C. Jennings, P. B. Jensen, J. Kermode, J. R. Kitchin, E. L. Kolsbjerg, J. Kubal, K. Kaasbjerg, S. Lysgaard, J. B. Maronsson, T. Maxson, T. Olsen, L. Pastewka, A. Peterson, C. Rostgaard, J. Schiøtz, O. Sch utt, M. Strange, K. S. Thygesen, T. Vegge, L. Vilhelmsen, M. Walter, Z. Zeng, K. W. Jacobsen. The Atomic Simulation Environment-A Python Library for Working with Atoms. *J. Phys.: Condens. Matter* **2017**, *29*, 273002.

[3] M. Pourbaix, *Atlas of Electrochemical Equilibria in Aqueous Solutions 2nd ed.*, National Association of Corrosion Engineers, Houston, Tex, **1974**.

[4] R. A. Flores, C. Paolucci, K. T. Winther, A. Jain, J. A. G. Torres, M. Aykol, J. M. J., J. K. Nørskov, M. Bajdich, T. Bligaard. Active Learning Accelerated Discovery of Stable Iridium Oxide Polymorphs for the Oxygen Evolution Reaction. *Chem. Mater.* **2020**, *32*, 5854–5863.

[5] E. Willinger, C. Massué, R. Schlögl, M. G. Willinger. Identifying Key Structural Features of IrO_x Water Splitting Catalysts. *J. Am. Chem. Soc.* **2017**, *139*, 12093–12101.

[6] K. A. Persson, B. Waldwick, P. Lazic, G. Ceder. Prediction of Solid-Aqueous Equilibria: Scheme to Combine First-Principles Calculations of Solids with Experimental Aqueous States. *Phys. Rev. B* **2012**, *85*, 235438.

[7] Y.-J. Lee, T. Lee, A. Soon. Phase Stability Diagrams of Group 6 Magnéli Oxides and Their Implications for Photon-Assisted Applications. *Chem. Mater.* **2019**, *31*, 4282–4290.

[8] R. Dronskowski, P. E. Bloechl. Crystal Orbital Hamilton Populations (COHP): Energy-Resolved Visualization of Chemical Bonding in Solids Based on Density-Functional Calculations. *J. Phys. Chem.* **1993**, *97*, 8617–8624.

[9] V. L. Deringer, A. L. Tchougréeff, R. Dronskowski. Crystal Orbital Hamilton Population (COHP) Analysis As Projected from Plane-Wave Basis Sets. *J. Phys. Chem. A* **2011**, *115*, 5461–5466.

[10] S. Maintz, V. L. Deringer, A. L. Tchougréeff, R. Dronskowski. Analytic Projection from Plane-

Wave and PAW Wavefunctions and Application to Chemical-Bonding Analysis in Solids. *J. Comput. Chem.* **2013**, *34*, 2557–2567.

[11] S. Maintz, V. L. Deringer, A. L. Tchougréeff, R. Dronskowski. LOBSTER: A Tool to Extract Chemical Bonding from Plane-Wave Based DFT. *J. Comput. Chem.* **2016**, *37*, 1030–1035.

[12] R. Nelson, C. Ertural, J. George, V. L. Deringer, G. Hautier, R. Dronskowski. LOBSTER: Local Orbital Projections, Atomic Charges, and Chemical-Bonding Analysis from Projector-Augmented-Wave-Based Density-Functional Theory. *J. Comput. Chem.* **2020**, *41*, 1931–1940.

[13] W. Tang, E. Sanville, G. Henkelman. A Grid-based Bader Analysis Algorithm without Lattice Bias. *J. Phys.: Condens. Matter* **2009**, *21*, 084204.

[14] J. Klimeš, D. R. Bowler, A. Michaelides. Chemical Accuracy for the Van Der Waals Density Functional. *J. Phys.: Condens. Matter* **2009**, *22*, 022201.

[15] J. Rossmeisl, Z.-W. Qu, H. Zhu, G.-J. Kroes, J. Nørskov. Electrolysis of Water on Oxide Surfaces. *J. Electroanal. Chem.* **2007**, *607*, 83–89.

[16] H. Hansen, J. Rossmeisl, J. Nørskov. Surface Pourbaix Diagrams and Oxygen Reduction Activity of Pt, Ag and Ni(111) Surfaces Studied by DFT. *Phys. Chem. Chem. Phys.* **2008**, *10*, 3722–3730.

[17] M. García-Melchor, L. Vilella, N. López, A. Vojvodic. Computationally Probing the Performance of Hybrid, Heterogeneous, and Homogeneous Iridium-Based Catalysts for Water Oxidation. *ChemCatChem* **2016**, *8*, 1792–1798.

[18] I. C. Man, H.-Y. Su, F. Calle-Vallejo, H. A. Hansen, J. I. Martínez, N. G. Inoglu, J. Kitchin, T. F. Jaramillo, J. K. Nørskov, J. Rossmeisl. Universality in Oxygen Evolution Electrocatalysis on Oxide Surfaces. *ChemCatChem* **2011**, *3*, 1159–1165.

[19] Z. Alexandra, A. Vitaly. Role of Defects in the Interplay between Adsorbate Evolving and Lattice Oxygen Mechanisms of the Oxygen Evolution Reaction in RuO_2 and IrO_2. *ACS Catal.* **2020**, *10*, 3650–3657.

[20] C. J. Cramer, *Essentials of Computational Chemistry: Theories and Models*, John Wiley & Sons, **2013**.

[21] A. A. Peterson, F. Abild-Pedersen, F. Studt, J. Rossmeisl, J. K. Nørskov. How Copper Catalyzes the Electroreduction of Carbon Dioxide into Hydrocarbon Fuels. *Energy Environ. Sci.* **2010**, *3*, 1311–1315.

[22] P. W. Atkins, J. De Paula, *Physical Chemistry*, **1998**.

[23] A. Jain, S. P. Ong, G. Hautier, W. Chen, W. D. Richards, S. Dacek, S. Cholia, D. Gunter, D. Skinner, G. Ceder, K. A. Persson. The Materials Project: A Materials Genome Approach to Accelerating Materials Innovation. *APL Materials* **2013**, *1*, 011002.

[24] S. Yagi, I. Yamada, H. Tsukasaki, A. Seno, M. Murakami, H. Fujii, H. Chen, N. Umezawa, H. Abe, N. Nishiyama, S. Mori. Covalency-Reinforced Oxygen Evolution Reaction Catalyst. *Nat. Commun.* **2015**, *6*, 1–6.

[25] A. L. Strickler, D. Higgins, T. F. Jaramillo. Crystalline Strontium Iridate Particle Catalysts for Enhanced Oxygen Evolution in Acid. *ACS Appl. Energy Mater.* **2019**, *2*, 5490–5498.

[26] W. Sun, Y. Song, X.-Q. Gong, L.-M. Cao, J. Yang. Hollandite Structure $K_{x\approx0.25}$ IrO_2 Catalyst with Highly Efficient Oxygen Evolution Reaction. *ACS Appl. Mater. Interfaces* **2016**, *8*, 820–826.

[27] Y. Lee, J. Suntivich, K. J. May, E. E. Perry, Y. Shao-Horn. Synthesis and Activities of Rutile IrO_2 and RuO_2 Nanoparticles for Oxygen Evolution in Acid and Alkaline Solutions. *J. Phys. Chem. Lett.* **2012**, *3*, 399–404.
